# PLGA/BGP/Nef porous composite restrains osteoclasts by inhibiting the NF-κB pathway, enhances IGF-1-mediated osteogenic differentiation and promotes bone regeneration

**DOI:** 10.1186/s13036-023-00354-8

**Published:** 2023-07-17

**Authors:** Feng Wu, Zhenxu Wu, Zhijun Ye, Guoqing Niu, Zhiliang Ma, Peibiao Zhang

**Affiliations:** 1 Foshan Hospital of Traditional Chinese Medicine/Foshan Hospital of TCM, Foshan, China; 2 Key Laboratory of Polymer Ecomaterials, Institute of Applied Chemistry, Chinese Academy of Sciences, Changchun, China

**Keywords:** Bioactive glass particle, Neferine, Osteoclast inhibition, Osteogenesis promotion, Bone regeneration

## Abstract

**Background:**

Novel bone substitutes are urgently needed in experimental research and clinical orthopaedic applications. There are many traditional Chinese medicines that have effects on bone repair. However, application of natural medicines in traditional Chinese medicine to bone tissue engineering and its mechanism were rarely reported.

**Results:**

In this study, the osteogenic ability of bioactive glass particles (BGPs) and the osteogenic and osteoclastic ability of neferine (Nef) were fused into PLGA-based bone tissue engineering materials for bone regeneration. BGPs were prepared by spray drying and calcination. Particles and Nef were then mixed with PLGA solution to prepare porous composites by the phase conversion method. Here we showed that Nef inhibited proliferation and enhanced ALP activity of MC3T3-E1 cells in a dose‐ and time‐dependent manner. And the composites containing Nef could also inhibit RANKL‐induced osteoclast formation (*p* < 0.05). Mechanistically, the PLGA/BGP/Nef composite downregulated the expression of NFATC1 by inhibiting the NF-κB pathway to restrain osteoclasts. In the other hands, PLGA/BGP/Nef composite was first demonstrated to effectively activate the IGF-1R/PI3K/AKT/mTOR pathway to enhance IGF-1-mediated osteogenic differentiation. The results of animal experiments show that the material can effectively promote the formation and maturation of new bone in the skull defect site.

**Conclusions:**

The PLGA/BGP/Nef porous composite can restrain osteoclasts by inhibiting the NF-κB pathway, enhance IGF-1-mediated osteogenic differentiation and promotes bone regeneration, and has the potential for clinical application.

**Supplementary Information:**

The online version contains supplementary material available at 10.1186/s13036-023-00354-8.

## Introduction

Bone tissue engineering has great potential in developing novel materials for bone regeneration. For the past few years, composites combining organic polymers (PLA, PLGA, etc.) with inorganic ceramic (hydroxyapatite, bioactive glass, etc.) attracted great interest [1–4]. In previous studies, poly(lactide-co-glycolide) (PLGA) was always chosen due to its good biocompatibility and biodegradability [1, 2].

Drug delivery is critical in bone tissue engineering. In previous studies, biopolymers and bioceramics were commonly used in the design of drug delivery systems for bone-related diseases [5–8]. Of all the materials in this category, bioactive glass is a great candidate for bone regeneration due to its biocompatibility, biodegradation, angiogenesis, bone conduction and bone-induced behavior [6]. Microspheres and particles, with the advantages of a large specific surface area, high loading capacity and controlled drug release, are the most common forms of drug delivery materials [7, 8].

The combination of biomaterials with signal factors (natural medicine, growth factor, active peptide, etc.) can significantly improve the biological activity of the materials [5, 9, 10]. In recent years, many compounds derived from natural plants have shown multiple biological properties, including antioxidant, anti-inflammatory and anticancer effects. Some of these compounds have also been shown to exert potent anti-osteoclasts and osteogenesis effects [11]. There are many traditional Chinese medicines that have effects on bone repair. However, application of natural medicines in traditional Chinese medicine to bone tissue engineering and its mechanism were rarely reported. Neferine (Nef) is a natural dibenzyl isoquinoline alkaloid extracted from the seeds of *lotus.* It has been used as a traditional Chinese medicinal plant to reduce inflammation and anxiety for over 1000 years. Modern medical research showed that Nef could moderate bleomycin-induced pulmonary fibrosis, suppress the proliferation of vascular smooth cells, and reduce the platelet aggregation rate. It was also found to have therapeutic effects on arrhythmias [12, 13]. Nef also regulates IGF-1R/Nrf2 signaling in H9c2 cardiomyocytes treated with doxorubicin. Doxorubicin induces a significant increase in mitochondrial superoxide in H9c2 cells, decreases the antioxidant capacity of cells, and inhibite the activation of IGF-1R signaling by inhibiting PI3K/Akt/mTOR. Neferine can activate IGF-1R signaling pathway, improve the antioxidant capacity of cells, and up-regulate the expression of PI3K/Akt/mTOR, and significantly inhibit mitochondrial superoxide production and autophagy [14]. Recently, neferine has been found to be a potential therapeutic application against osteolytic bone conditions such as osteoporosis. Chen et al. reported the inhibition of Nef on RANKL-induced osteoclast formation. In addition, Nef can effectively reduce the bone resorption activity of mature osteoclasts. Mechanistically, Nef inhibits RANKL-induced activation of NF-κB signaling pathway, which in turn downregulates the expression of NFATC1, resulting in down-regulation of related osteoclast marker genes. Meanwhile, Nef enhances the differentiation and bone mineralization activity of MC3T3-E1 cells. These results indicate that Nef is a potential signaling factor in bone tissue engineering [15].

**Fig. 1:**
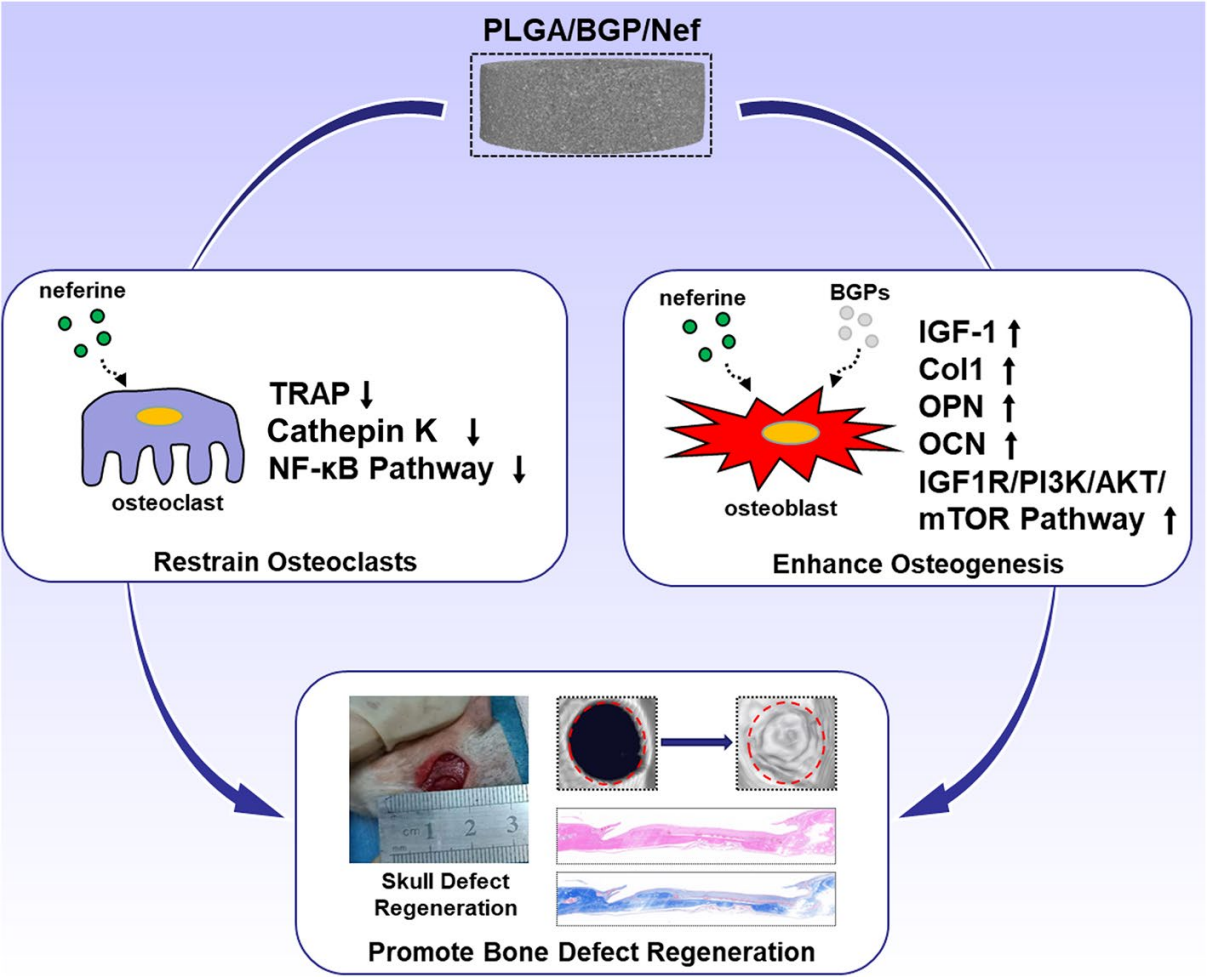
Schematic diagram of PLGA/BGP/Nef porous composite restraining osteoclasts by inhibiting the NF-κB pathway, enhancing IGF-1-mediated osteogenic differentiation and promoting bone regeneration

In this research, bioactive glass particles (BGPs) were prepared by spray drying and calcination. Particles and Nef were then mixed with PLGA solution to prepare porous composite scaffolds by the phase conversion method. The aim of this study was to improve the osteoclast-inhibition and osteo-induction ability of composites through the slow release of Nef. The osteo-differentiation of MC3T3-E1 cells and the osteoclast differentiation of RAW264.7 cells were explored to investigate their effectiveness. In addition, the underlying mechanisms of osteogenesis and osteoclast-inhibition by this composite were briefly researched in this study. Subsequently, the osteogenic repair ability of the material was evaluated through repair experiment of the rat skull defect model (Fig. 1).

## Materials and methods

### Preparation of BGPs

20 g citric acid was added to 300 mL deionized water and stir until completely dissolved. After that, 125 g of ethyl orthosilicate, 14.6 g of triethyl phosphate and 85 g of calcium nitrate tetrahydrate were successively added to the stirred solution at an interval of 30 min. After continuous stirring for 3 h, the solution was left standing at room temperature overnight and protected from light. Then, the dry gel particles were prepared by spray drying. The collected dry gel particles were calcined to obtain bioactive glass particles. Calcination conditions: under the condition of full air circulation, slowly heating to 500℃, then rapidly heating to 850℃ and holding for 2 h, finally cooling to room temperature naturally.

### Fabrication of the PLGA/BGP/Nef Composites

PLGA/BGP/Nef composites containing 20 wt% Nef/BGPs were obtained via phase inversion. Different contents of Nef and BGPs were uniformly dispersed in the PLGA solution in NMP by stirring. The suspension was then frozen at -80 °C for 3 h. Afterwards, the freeze-formed bracket was placed in pure water for replacement, and the water was changed every 6 h for 2 days. After replacement, the composites were removed and placed in a freeze-drying machine for 12 h to obtain dry PLGA/BGP/Nef composite scaffolds. The content of Nef is expressed as the concentration of Nef in the initial solution.

### Characterization of the bioactive glass particles

Fourier transform infrared (FT-IR, Watford, UK) spectroscopic analysis was carried out to analyze the chemical composition of the BGPs. Environmental scanning electron microscopy (ESEM, XL30 FEG, Philips, Germany) was used to observe the micromorphology and size of the BGPs. The diameter distribution of the nanoparticles was analyzed using ImageJ. The drug loading and releasing capacity of BGPs was determined using BSA as a model. BGPs (100 mg) were immersed in 5 mL of a model protein solution of 200 μg/mL in PBS (pH 7.2). 100 μL of supernatant were taken every 10 min for concentration testing by the BCA method. For the release test, 60 min later, the remaining supernatant was exchanged with 5 mL PBS (pH 7.2). Then, 100 μL supernatant was taken every 10 min for the concentration test by the BCA method.

### Characterization of the BGP/PLGA/Nef Composites

The micromorphology and microstructure of scaffolds was observed by ESEM (XL30 FEG, Philips) and micro-CT (Bruker, SkyScan1172, Germany). The porosity of the scaffolds was computed using CTAn software based on the micro-CT data. FT-IR (Watford, UK) spectroscopic analysis was carried out to analyze the chemical composition of the composites. Then, 0.5 g of sterile sample was added to a centrifuge tube containing 30 mL of a sterile PBS solution, and the tube was placed in a shaker at 37 °C. A volume of 5 mL of the supernatant was extracted on time for Nef release measurement by ultraviolet detection, and 5 mL of a sterile PBS solution was added to the tube. After 10 min, 30 min, 1, 2, 4, 8, 16, and 24 h, the collected solution was extracted thrice with equal volumes of ethyl acetate and then evaporated using a rotary evaporator and redissolved in equal volumes of methanol. The Nef concentration was detected by a UV spectrophotometer at 270 nm and calculated according to the standard curve.

### Cell culture

MC3T3-E1 and RAW264.7 cells (Shanghai Institutes for Biological Sciences, Chinese Academy of Sciences) were used in this research. The Dulbecco’s minimum essential medium (DMEM, HyClone, USA) containing 10% FBS (Gibco, USA), 10 mM HEPES (Sigma), 100 mg/L streptomycin (Sigma), and 63 mg/L penicillin (Sigma) was prepared. Cells were seeded on the composite films and cultured at 37 °C in 5% CO_2_. For RAW264.7 cells, RANKL (50 ng/ml) was added in medium throughout the culture. Proliferation was detected by CCK-8 assay kit (Solarbio, Beijing). The detection method of alkaline phosphatase activity is as follows: the cell culture-medium was discarded and the cells were washed with PBS for twice. 200 μL of cell lysis solution was added into each well, followed by freeze-thawing for three times at -80℃. After centrifuged at 12,000 rpm, the supernatant was collected. The ALP and total protein in the supernatant were detected by the alkaline phosphatase assay kit (Solarbio, Beijing) and BCA detection kit (Thermo), respectively. The relative ALP activity was calculated as follow:

$$\mathrm{Relative}\;\mathrm{ALP}\;\mathrm{activity}\hspace{0.17em}=\hspace{0.17em}\mathrm{ALP}\;(\mathrm{OD}\;405)/\;\mathrm{BCA}(\mathrm{OD}\;562)$$

Total RNA was extracted using TRIzol Reagent (Invitrogen). And the reverse transcription was governed using PrimeScript RT Reagent Kit with gDNA Eraser (Perfect Real Time, TaKaRa). All samples were performed in triplicates in 8 striped optical tube (Axygen) using qPCR SYBR Green Mix Kit (Stratagene). All the primers using in this study were listed in Table 1. Specificity of listed oligonucleotides were checked by Basic Local Alignment Search Tool (BLASTN) against the homo RefSeq RNA database at NCBI. The Catk level in RAW264.7 cells culture medium was detected using an antibody-sandwich enzyme-linked immunosorbent assay (ELISA) method (Mouse Ctsk ELISA Kit, Finebio, Wuhan). The detection range is from 15.625 to 1000 pg/mL. All samples were diluted 100 times with dilution buffer before testing.

**Table 1:** Primers used in qRT-PCR

Gene	Forward primer sequence (5’-3’)	Reverse primer sequence (3’-5’)
Col1	CCCAGCGGTGGTTATGACTT	TCGATCCAGTACTCTCCGCT
Runx2	GCCAGTAATCTTCGTGCCAG	TAGTGAGCTTCTTCCTGGGGA
OPN	CCAGCCAAGGACCAACTACA	AGTGTTTGCTGTAATGCGCC
OCN	AAGCAGGAGGGCAATAAGGT	TTTGTAGGCGGTCTTCAAGC
IGF-1	GTGTGCCTCCCATACTGCTT	TGCTGATTTTCCCCATCGCT
IGF-1R	TTTCCACTCCGCATTTCTGC	CGTCCAAAAACAAGAGCGCA
CatK	CTTCCAATACGTGCAGCAGA	TCTTCAGGGCTTTCTCGTTC
GAPDH	CTTGTGCAGTGCCAGCCTC	GATGGTGATGGGTTTCCCGT

### Preparation of protein extract and immunoblotting

Total protein was extracted by using RIPA buffer (Solarbio, Beijing). Protein was quantified using the BCA method. And equal amounts of protein were loaded in a 12% SDS‒PAGE gel for separation through electrophoresis. Then the separated proteins were blotted onto an activated PVDF membrane at 150 V for 1 h. After blocked in 5% nonfat milk powder in TBST for 1 h at room temperature, the membrane was rinsed with TBST three times for 5 min each. Then, the blots were incubated with a specific primary antibody at 4℃ overnight. Subsequently, the samples were rinsed three times followed by incubation HRP-conjugated secondary antibody for 1 h at room temperature. Finally, the blots were visualized using ECL substrate, and images of the blots were analyzed densitometrically using ImageJ software.

### Animals and experimental protocol

Eight female Wistar rats (8 weeks, 180–220 g) were used in this study. All animal experiments were approved by the Institutional Animal Care and Use Committee of Changchun Institute of Applied Chemistry, CAS. Based on the in *vitro* experiments, the composite of PLGA/BGP/25N was selected as PLGA/BGP/Nef for animal experiments.

The experimental animals were in narcotism by injecting 3% pentobarbital sodium into the cavum abdominis (30 mg/kg). A posterior incision approximately 20 mm in length along the centerline was made from the midpoint of the two posterior canthi. The subcutaneous tissue was separated to fully expose the cranial parietal bone. Two circular defects of 5 mm in diameter were drilled on both sides of the parietal bone to the depth of the dura mater. The 16 defects were evenly divided into four groups. Different samples were implanted in the defect area, and the nonimplant group was used as the blank control group. Samples from the same group were assigned to different rat. A total of 80,000 IU/day penicillin sodium was injected intramuscularly for 5 days after the operation.

Micro-CT (Perkin Elmer, QuantumGX2-2-E) was performed every two weeks to observe the repair effect during the process. The regeneration coverage rate was detected by ImageJ. Bone volume fraction (BVF, BV/TV) at the defect site was detected by CTAn. The final repair effect was evaluated by tissue section staining (H&E, Masson and TRAP).

### Statistical analysis

Unless otherwise stated, the measurements were performed in three or four independent replicates. The obtained data are expressed as the mean ± standard deviation. Statistical analysis was performed using ANOVA with SPSS 26.0. The differences were considered statistically significant for *p* < 0.05.

## Results

### Characterization of the BGPs

#### Morphology

**Fig. 2:**
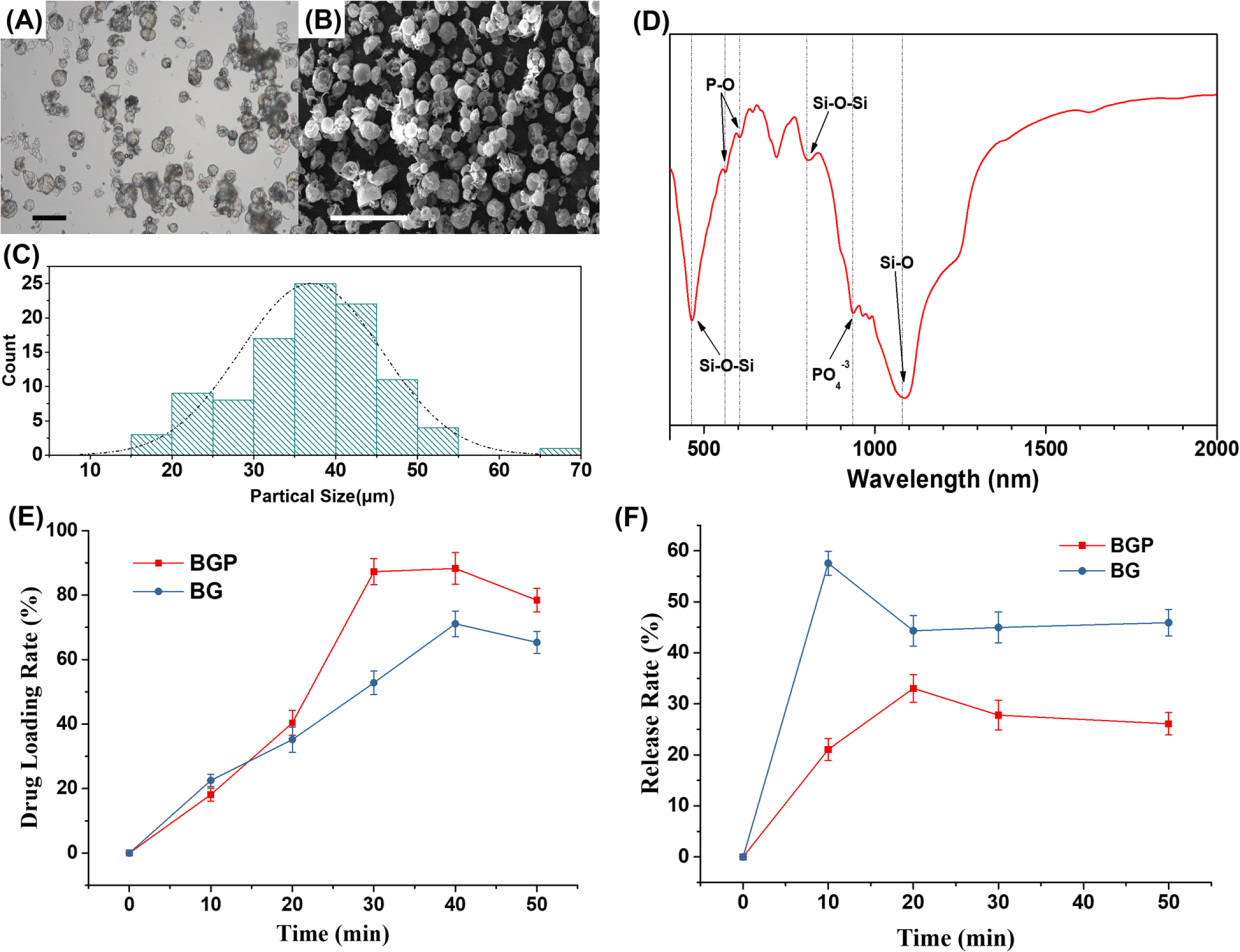
Characterization of the BGPs. The optical microscope (**A**) and SEM photographs (**B**) of BGPs. **C** the particle size distribution of BGPs. **D** the FT-IR spectra of the BGPs. BSA loading (**E**) and release (**F**) of BGP and BG. Bar = 100 μm (**A**) or 200 μm (**B**)

The optical microscope and SEM photographs of BGPs are shown in Fig. 2(A) and Fig. 2(B). The bioactive glass particles were spherical. The inside of the spheroid was hollow. Figure 2(C) shows the particle size distribution of BGPs. The particle size of BGPs mainly ranged from 15 to 55 μm. BGPs with particle sizes between 30 and 50 μm accounted for more than 75% of the total BGPs. The particle size follows a normal distribution. This indicated that the BGPs had good homogeneity.

#### FT-IR Spectra

The FT-IR spectra of the BGPs are shown in Fig. 2(D). The peak near 1090 nm is the stretching vibrational peak of Si–O. The peaks near 465 nm and 800 nm are the bending vibrational peak of Si–O-Si and the vibration peak of silica tetrahedral, respectively. The peaks near 560 nm and 605 nm are the bending vibrational peaks of P-O. All peaks are the characteristic peaks of the infrared spectrum of bioactive glass.

#### BSA loading and release

In this part, bioactive glass particles (BG) prepared by sol–gel method and grinding were prepared as contrast. The SEM photo is performed in Fig. S1(A). The BG is not mesoporous and presents the appearance of large particles formed by agglomeration of nanoparticles. As shown in Fig. 2(E), the adsorption quantity of BSA to BG and BGP increased gradually over time. After 30 min, the protein adsorption rate of BGP group peaked the value of 88.2%, while the maximum protein adsorption rate of BG was 65.1% at 45 min. The protein adsorption rate of BGP was higher than that of BG. The BSA release is shown in Fig. 2(F). A protein burst occurred in the BG group. The release rate first rapidly rose to 58% in 10 min, then fell back to approximately 45% and remained constant. In contrast, the release rate of the BGP group gradually increased to 33% within 20 min, then dropped below 30% and remained stable. BGP has advantages over BG in controlling protein release.

### Characterization of the composites

#### Effect of Different Nef Contents on MC3T3-E1 Cells

**Fig. 3:**
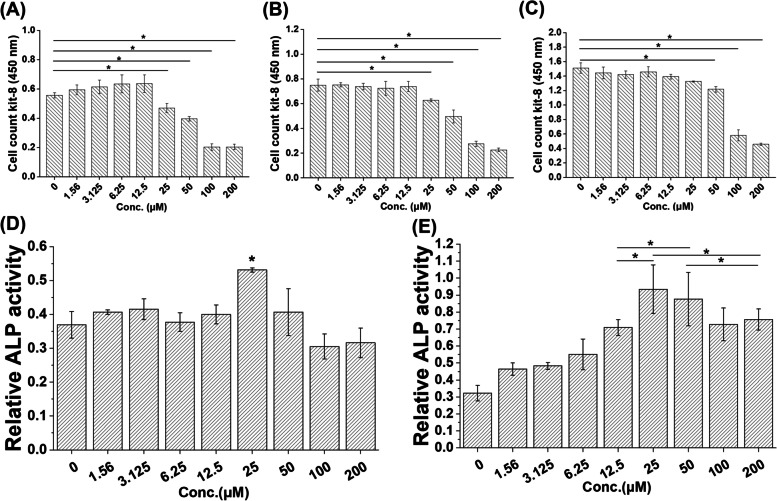
Effect of different Nef contents on MC3T3-E1 cells. The proliferation (CCK-8) of MC3T3-E1 cells on composites of different Nef contents on day 1(**A**), 3(**B**), and 7(**C**). Relative ALP activity of MC3T3-E1 cells on composites of different Nef contents on day 3 (**D**) and 7 (**E**). *p < 0.05, **p < 0.01

To determine the applied dosage of Nef, the proliferation and alkaline phosphatase (ALP) activity of MC3T3-E1 cells planted on composite membranes containing different Nef contents were detected. According to the results in Fig. 3(A-C), the cells in all groups continuously proliferated with culture. Nef at low concentrations (1.56 μM ~ 12.5 μM) had no obvious toxicity to cells, while Nef at concentrations over 25 μM significantly inhibited cell proliferation (*p* < 0.05). However, at day 7 of cell culture, the inhibitory effect of the 25 μM and 50 μM treatments on cell proliferation decreased, and there was no significant difference between the 25 μM and control groups (*p* > 0.05). And the comparison between different time points showed that the cells of PLGA/BGP/25N and PLGA/BGP/50N groups showed a well proliferation rate with culture.

The results of ALP activity are shown in Fig. 3(D-E). Composites of PLGA/BGP/25N could promote the ALP activity of MC3T3-E1 cells on day 3 (*p* < 0.05). All composites containing Nef could promote the ALP activity of MC3T3-E1 cells on day 7 (*p* < 0.05). However, the ALP activity of the PLGA/BGP/25N and PLGA/BGP/50N groups was the highest and significantly higher than other groups (*p* < 0.05). Based on the results of proliferation and ALP activity assays, Nef concentrations of 25 μM and 50 μM were selected for further research in this study.

#### Morphology

**Fig. 4:**
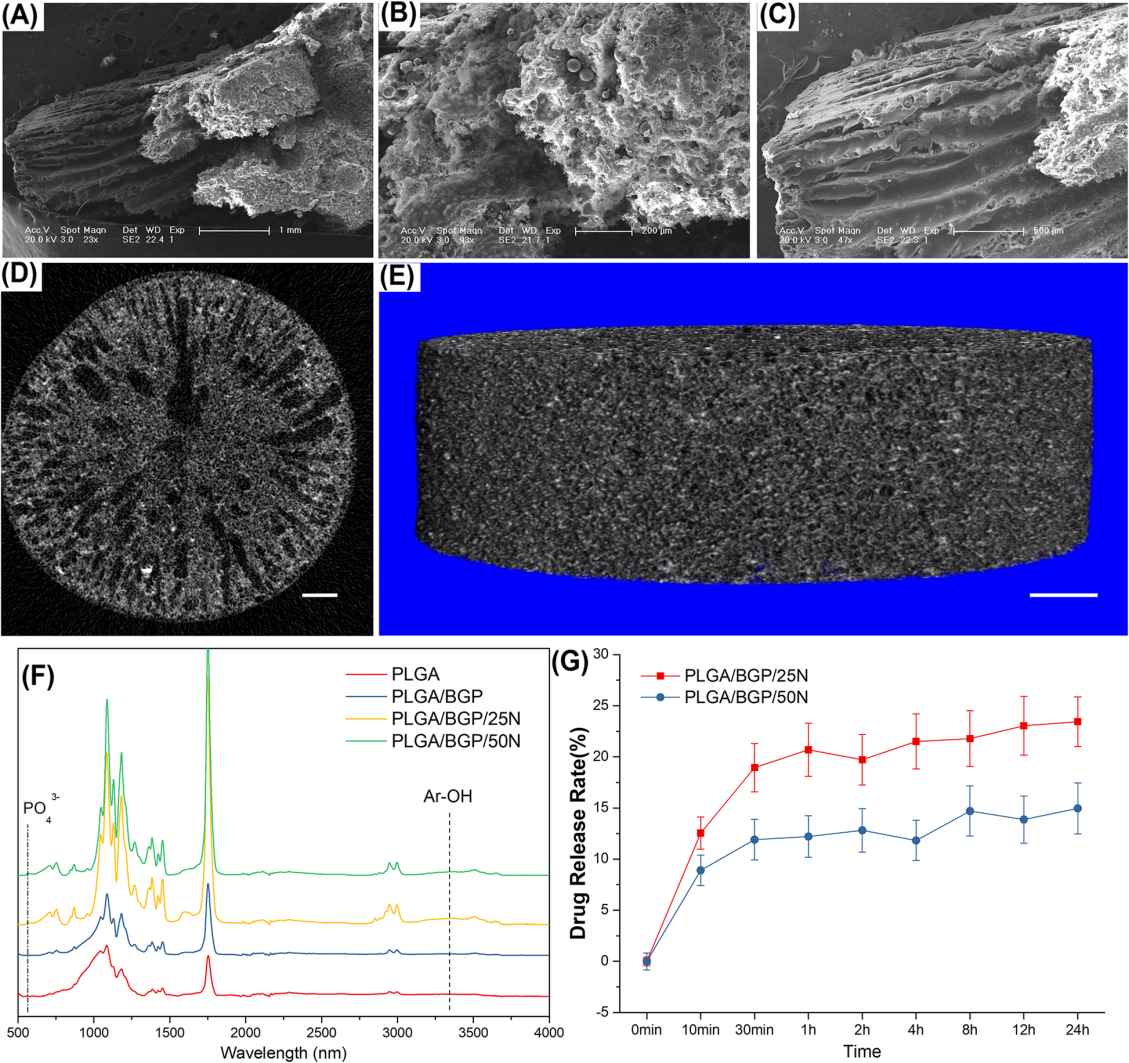
Physical and chemical characterization of the composites. **A**-**C** SEM photographs of PLGA/BGP composite in different magnifications and positions. The scale size is shown in the figure. **D** The single layer scan and (**E**) 3D reconstruction of PLGA/BGP composite based on micro-CT results. Bar = 1 mm. **F** The FT-IR spectra of the composites. **G** The release behavior of Nef in 24 h

The microscopic structures observed by ESEM and micro-CT are shown in Fig. 4(A-E). The whole composite material presents a loose and porous structure. The porosity is up to 86.35%. The pore structure of the central part is different from that of the peripheral part. The center part presents a homogeneous honeycomb structure. Radial vascular channels with pipe diameters ranging from 100 μm to 150 μm exist around the periphery, extending from the center to the edge of the composite. A large amount of BGP is evenly distributed in the composite.

#### FT-IR Spectra

The FT-IR spectra of the composites are shown in Fig. 4(F). In addition to the characteristic peaks of the infrared spectrum of bioactive glass, the characteristic peak of the phenolic hydroxyl group can be seen near 3280 nm, which indicated the existence of Nef. Because of the low content of Nef in the material, the characteristic peak is low. So that it is hard to be observed. In order to point out the characteristic peak of Ar-OH, the FT-IR spectra of the composites between 3240 and 3300 nm was drawn in Fig. S2. A distinct characteristic peak was observed near 3280 nm in PLGA/BGP/25N combined PLGA/BGP/50N group.

#### In vitro* Nef Release Profile*

The release behavior of Nef in 24 h is shown in Fig. 4(G). It exhibited an initial burst release of Nef within the first half hour, which was determined to be 18.7% in the PLGA/BGP/25N group and 12.2% in the PLGA/BGP/50N group. Then, the drug showed sustained release, and the cumulative release of Nef reached 23.4% and 14.9% and was still continuing to be released slowly.

#### Anti-osteoclastogenic effect of composites

**Fig. 5:**
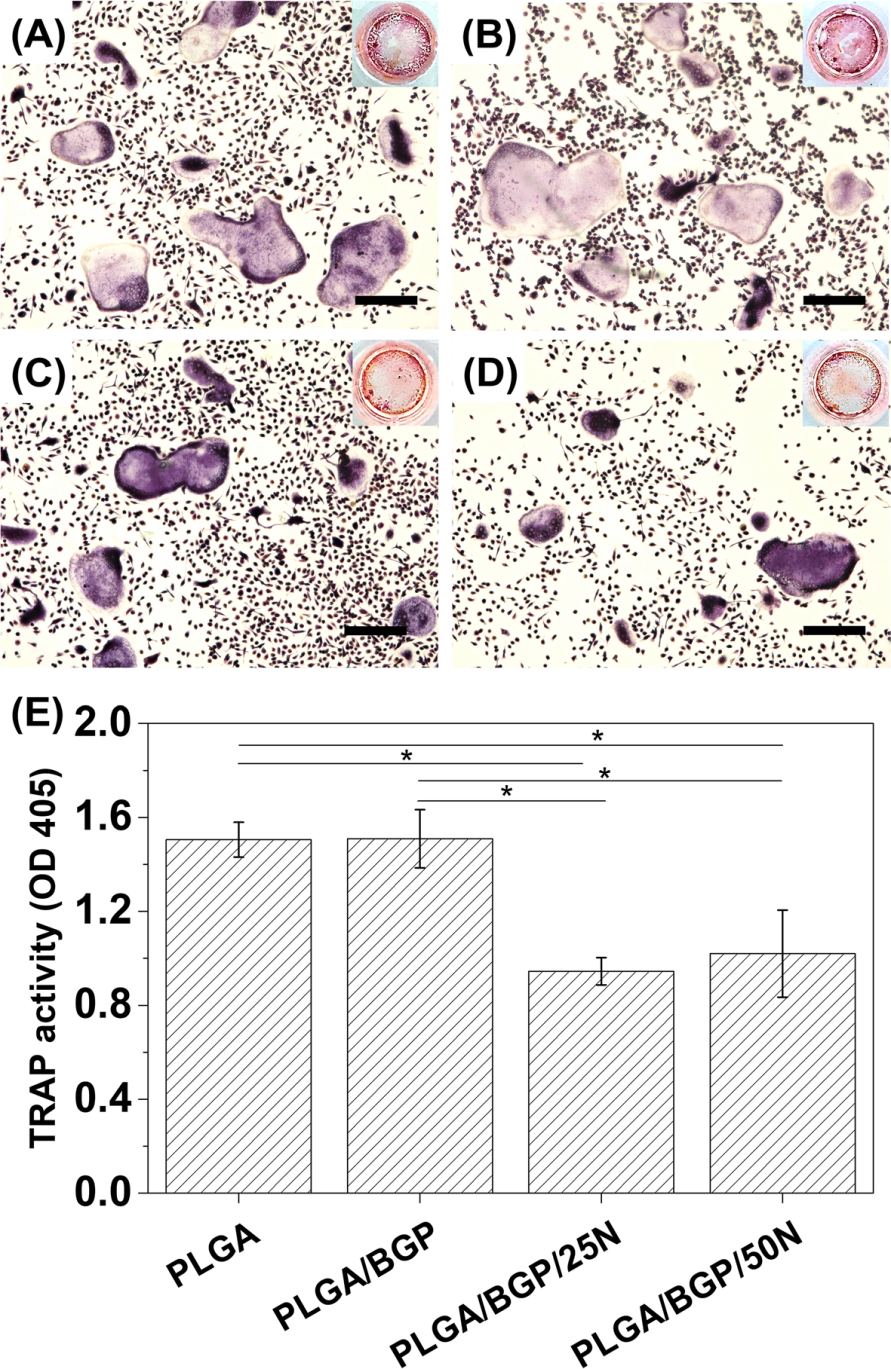
Anti-osteoclastogenic effect of composites. TRAP staining of osteoclasts after RANKL stimulating in groups of PLGA (**A**), PLGA/BGP (**B**), PLGA/BGP/25N (**C**), and PLGA/BGP/50N (**D**). Bar = 100 μm. **E**The result of TRAP activity assay in different groups. *p < 0.05

The effects of the composites on osteoclast differentiation were examined. M‐CSF‐dependent macrophages were cotreated with RANKL and cultured on different composites until large multinucleated “pancake”‐shaped osteoclasts were observed in the RANKL‐treated controls. The fixed cells were further stained for TRAP activity. As demonstrated in Fig. 5, Nef inhibited the total number of TRAP‐positive osteoclasts formed in response to RANKL. The TRAP activity was further detected by Tartrate Resistant Acid Phosphatase Assay Kit (Beyotime, Beijing). According to result of TRAP activity assay, the TRAP activity in the Nef groups was significantly lower than that in the control and PLGA/BGP groups (*p* < 0.05). The results show that the composites containing Nef can inhibit RANKL‐induced osteoclast formation.

### *Gene expression analysis *via* qRT‒PCR*

**Fig. 6:**
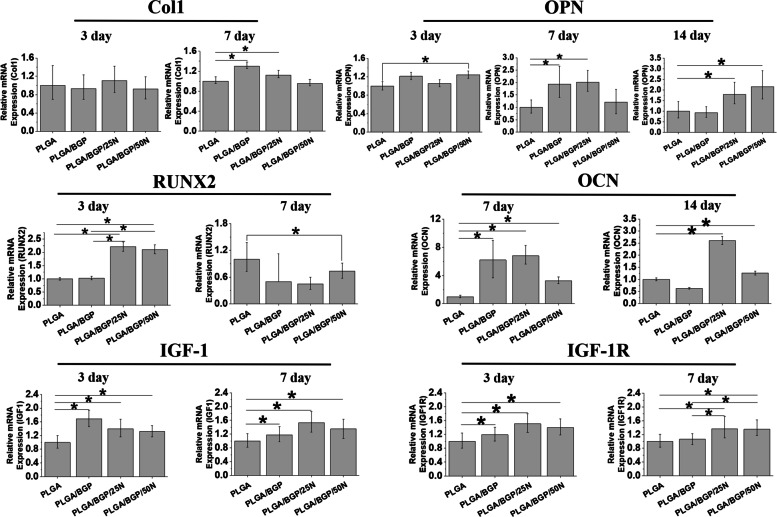
The results of gene expression analysis via qRT‒PCR in MC3T3-E1. *p < 0.05

To explore the effect of different composites on the osteogenic differentiation of MC3T3-E1 cells at the molecular level, the real-time quantitative PCR (qRT-PCR) was used to quantitatively measure the osteogenic differentiation-related target genes Runt-related transcription factor-2 (RUNX2), type I collagen (Col1), Osteopontin (OPN), and Osteocalcin (OCN). As shown in Fig. 6, MC3T3-E1 cells were cocultured with different composites for 3 days, 7 days and 14 days. For Col1, there was no significant difference between each group at day 3. After 7 days of culture, compared with the PLGA group, the expression levels of Col1 genes were increased significantly in the PLGA/BGP and PLGA/BGP/25N groups. The expression levels in PLGA/BGP/50N were similar to those in the control group. In addition, with the increase in Nef content, the expression level of Col1 decreased gradually.

The composites containing Nef promoted the expression levels of RUNX2 at day 3. On day 7, RUNX2 expression was significantly higher in the PLGA group than in the other groups. However, RUNX2 expression in the PLGA/BGP/50N group was still higher than that in the PLGA/BGP group.

Gene expression of OPN was measured at days 3, 7 and 14. There was no significant difference between the groups on day 3. After 7 days of culture, compared with the PLGA group, the expression levels of OPN genes were increased significantly in the PLGA/BGP and PLGA/BGP/25N groups. The expression levels of OPN in the PLGA/BGP/50N group were also higher than those in the PLGA group. However, the difference was not statistically significant. The results of day 14 showed that the expression levels of OPN in the PLGA/BGP/25N and PLGA/BGP/50N groups were obviously promoted when compared to the other two groups. The expression level of OPN increased gradually with increasing Nef content.

Gene expression of OCN was measured at days 7 and 14. At 7 days, the expression of OCN in the PLGA/BGP, PLGA/BGP/25N and PLGA/BGP/50N groups was obviously upregulated compared with that in the PLGA group. The expression level of PLGA/BGP/25N was the highest. At 14 days, the OCN expression in the PLGA/BGP/25N group was still highest. The expression levels of OCN in the PLGA/BGP/25N and PLGA/BGP/50N groups were obviously upregulated compared to those in the other two groups.

IGF-1 plays an important role in bone repair. IGF-1R is a key receptor for IGF-1. In this study, the gene expression of IGF-1 and IGF-1R in MC3T3-E1 cells was detected at 3 and 7 days. The IGF-1 expression level in the PLGA group was significantly lower than that in the other three groups at each timing. However, at day 3, the levels of IGF-1 in the Nef-containing groups were lower than those in the PLGA/BGP group. Conversely, at 7 days, IGF-1 expression was higher in the Nef-containing groups than in the PLGA/BGP group. IGF-1R expression in the PLGA/BGP/25N and PLGA/BGP/50N groups was obviously upregulated compared with that in the PLGA group at days 3 and 7. The expression level of the PLGA/BGP group was higher than that of the PLGA group only at 3 days and was the same as that of the PLGA group at 7 days.

**Fig. 7:**
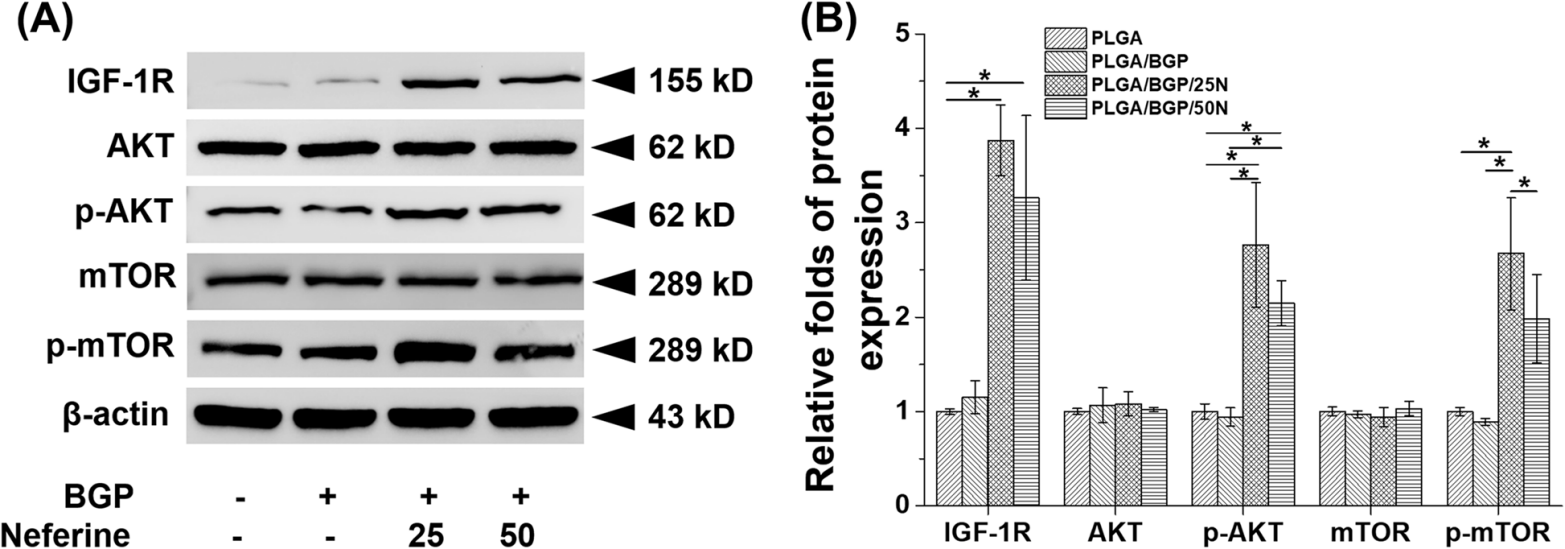
The results of protein expression by western blotting in MC3T3-E1. **A** the bands of proteins; **B** Systematic analysis of the protein bands, *p＜0.05

The expression of CatK was shown in Fig. 7(A). CatK is a cysteine protease with the highest expression and the strongest bone lytic activity in osteoclasts and is a key enzyme in the process of bone resorption. In this study, the expression level of CatK in RANKL‐treated osteoclasts was significantly downregulated in the PLGA/BGP/25N and PLGA/BGP/50N groups compared with the PLGA group at both 3 and 7 days. The same trend was observed in extracellular secretion of CatK tested by ELISA (Fig. 7(B, C)).

#### Protein expression by western blotting

**Fig. 8:**
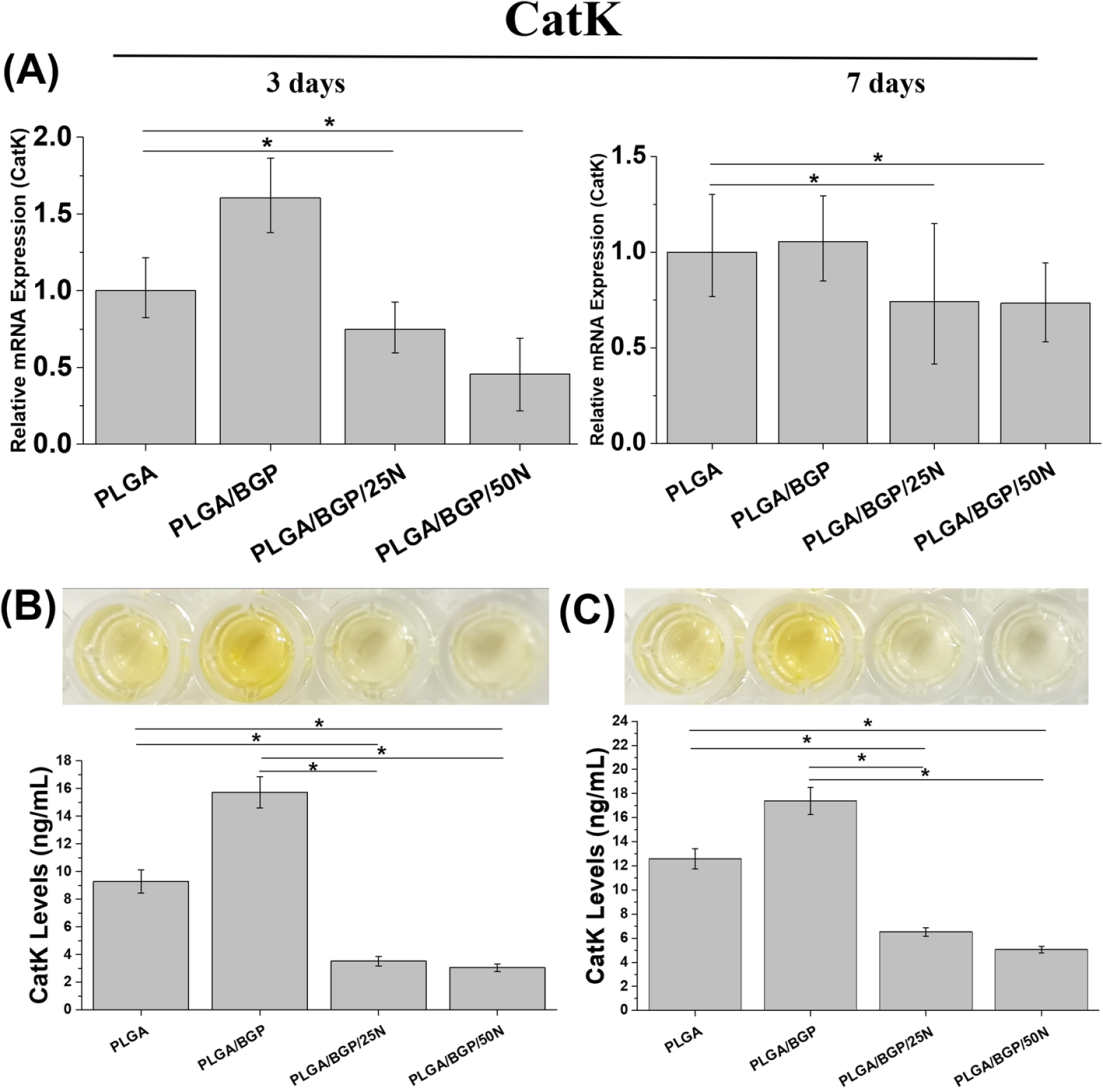
(**A**) gene expression analysis of CatK via qRT‒PCR in RANKL‐induced osteoclast. The Catk level of 3 d (**A**) and 7 d (**B**) in RAW264.7 cells culture medium detected by antibody-sandwich ELISA method. *p＜0.05

In this study, MC3T3-E1 and RANKL‐induced osteoclasts cultured on composite membranes for 7 days were collected. For MC3T3-E1 cells, IGF-1-mediated osteogenic differentiation pathway proteins were detected. Western blotting (Fig. 8) results indicated that, compared with the PLGA and PLGA/BGP groups, the amount of IGF-1-mediated osteogenic differentiation pathway proteins (IGF-1R, p-AKT and *p*-mTOR) produced in the Nef-containing groups increased, and the expression levels in PLGA/BGP/N25 were the highest.

**Fig. 9:**
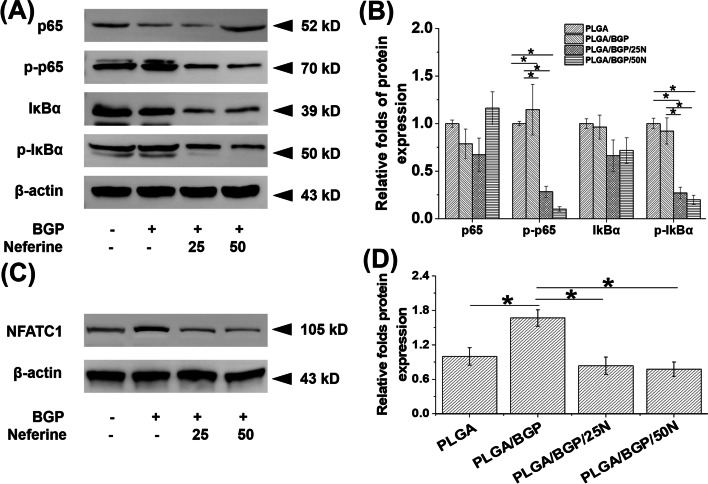
The results of protein expression by western blotting in RANKL‐induced osteoclast. **A**,**C** the bands of proteins; **B**,**D** Systematic analysis of the protein bands, *p < 0.05

For RANKL‐induced osteoclasts, NF-κB pathway-related pathway proteins and the osteoclast differentiation-related transcription factor NFATC1 were detected (Fig. 9). Compared with the PLGA and PLGA/BGP groups, the phosphorylation of NF-κB pathway-related pathway proteins (p-p65 and p-IκBα) were reduced in the Nef-containing groups decreased. The expression of NFATC1 was also significantly inhibited (*p* < 0.05).

#### Repair effect of skull injury

**Fig. 10:**
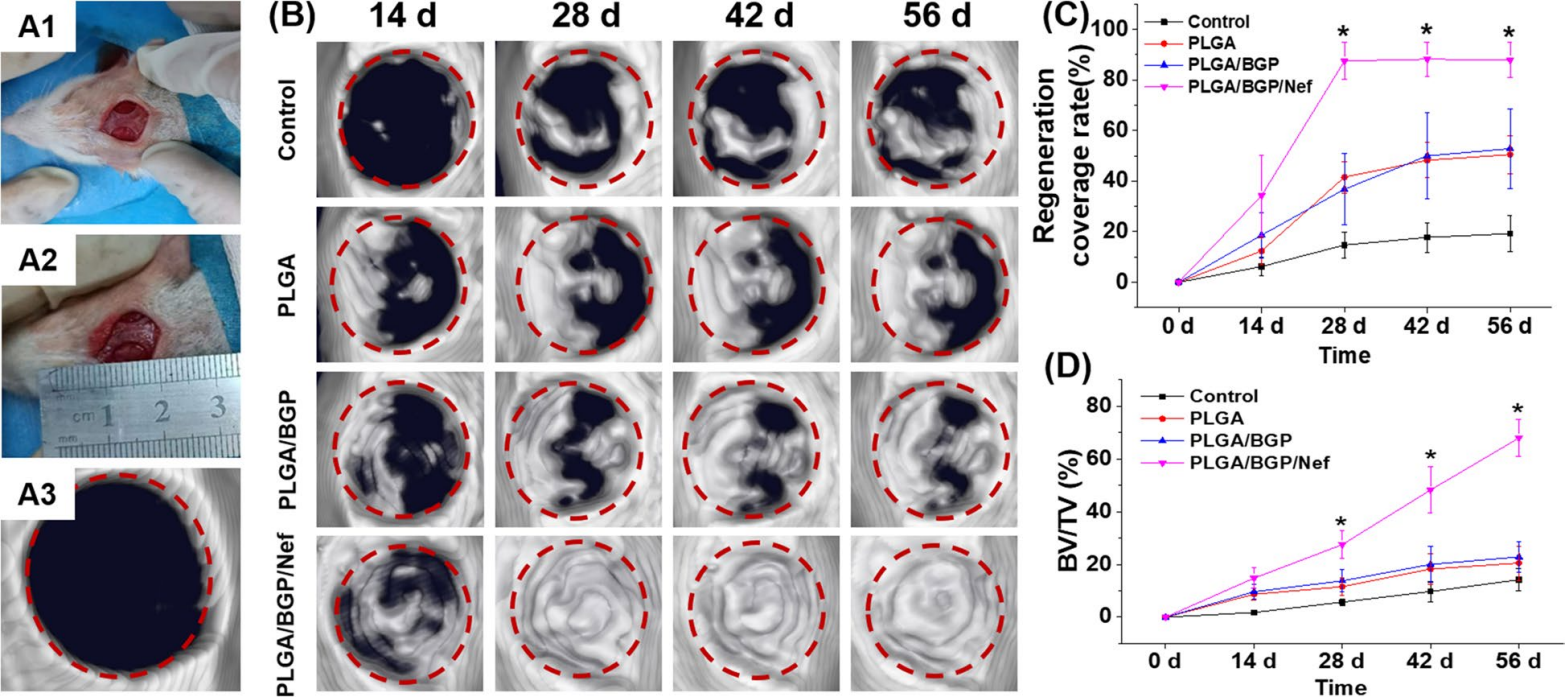
The changes in skull defects over time observed by micro-CT. (A1-A2) Model building of skull defect; (A3) The 3D reconstruction of postoperative defect site by micro-CT; **B** Micro-CT images of the rat calvarial bone at different time points; **C** Regeneration coverage rate and **D** bone volume fraction (BVF, BV/TV) at the defect site. *p < 0.05

The changes in skull defects over time observed by micro-CT are shown in Fig. 10. The defects in each group were gradually covered by new bone tissue. The PLGA/BGP/Nef group had the highest regeneration coverage at all time points (*p* < 0.05), exceeding 80% coverage at day 28. The regeneration coverage rate was approximately the same in the PLGA and PLGA/BGP groups, reaching approximately 50% after 56 days, while that of the control group was less than 20%. The BVF at the defect site of PLGA/BGP/Nef was also significantly higher than that of the other groups at each time point (*p* < 0.05), up to approximately 70% at day 56. The BVFs of the PLGA/BGP group, PLGA group, and control group decreased in sequence. However, the difference between the PLGA/BGP group and PLGA group was not significant.

**Fig. 11:**
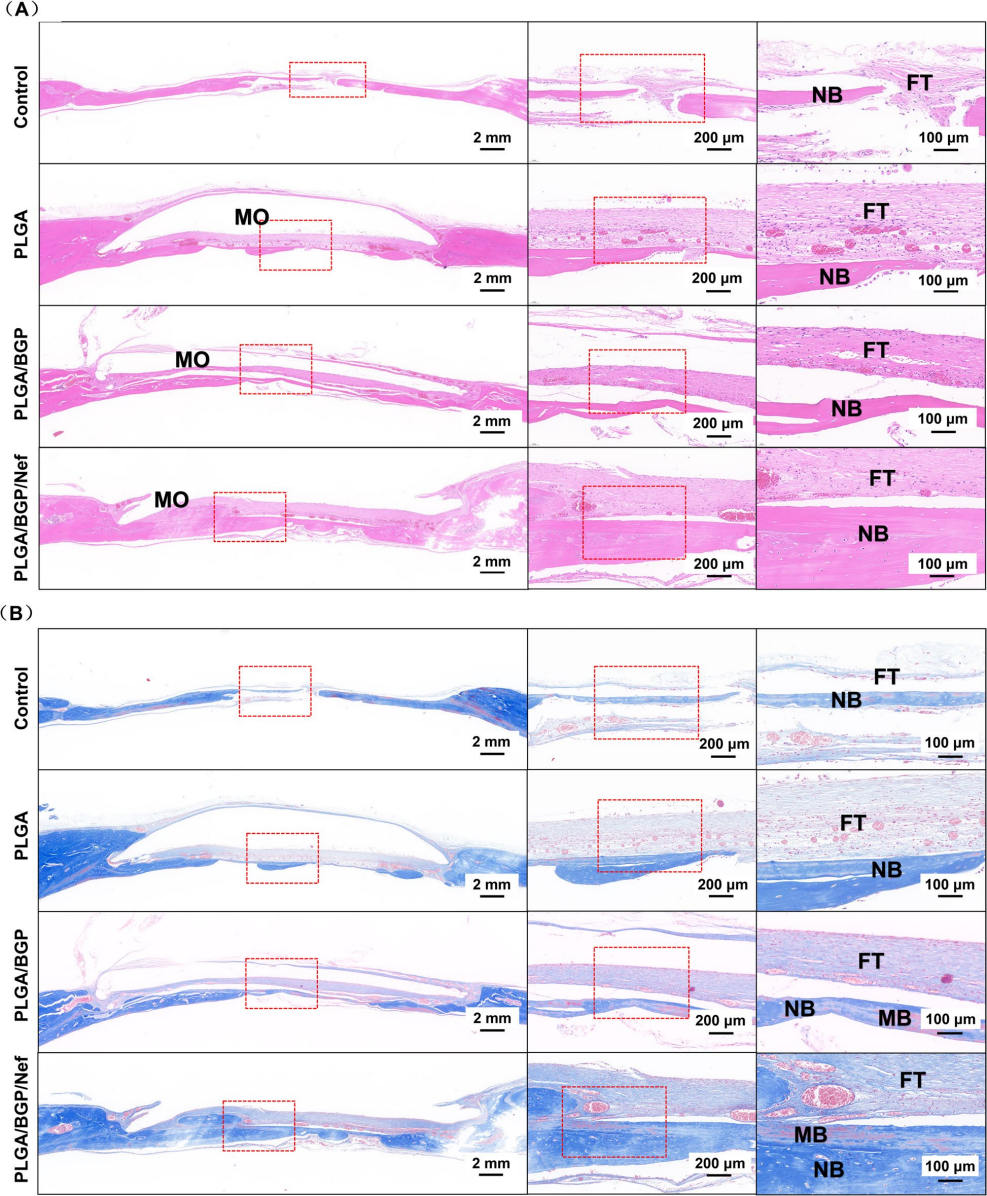
H&E staining (**A**) and Masson staining (**B**) in rat calvarial bone defects at 8 weeks after surgery. FT: fibrous tissue; MO: material occupation; NB: new bone; MB: mature bone

H&E and Masson staining were used to highlight typical features in tissue sections. Figure 11(A) shows the results of H&E staining. Connective tissue was formed along the occupation of material. The occupying part of the new bone tissue grew and gradually covered the defect, while the defect did not form a complete osseous connection in the control group. The implants act as a “bone bridge”. Especially in the PLGA/BGP/Nef group, the new bone formed thicker calli, and the repair effect was significantly better than that in the other groups. Figure 11(B) shows the results of Masson staining. Most of the defect sites in each group had been covered with newborn tissue. Scattered mature bone tissue stained red can also be observed inside the blue new bone. The thickness of new bone in the PLGA/BGP/Nef group was thicker than that in the other groups. There was also more red staining in the new bone site of this group. The results further confirmed that PLGA/BGP/Nef could enhance the effect of bone defect repair. Figure S7 shows the TRAP staining results in rat calvarial bone defects. Red staining along the bone surface is one of the main characteristics of TRAP staining. A large number of red staining areas on the surface of the bone were observed in PLGA/BGP group, while there were only few red staining areas in the other three groups. This is consistent with previous data in RT-PCR and westernblotting.

## Discussions

The development of new bone tissue engineering substitutes is one of the most interesting research directions in the treatment of bone defects. Novel bone regeneration composites composed of diverse polymers, bioceramics and bioactive molecules have different biocompatibilities, mechanical properties and osteogenic activities. Therefore, the design and development of novel bone substitutes are urgently needed in experimental research and clinical orthopaedic applications. In this study, PLGA was used as the substrate material. Bioactive glass particles (BGPs) were compounded with PLGA to prepare the PLGA/BGP composite by a phase exchange method. Bioactive glass has long been used to repair hard tissue in the human body because it can bond to bone tissue. Bioactive glass is an amorphous material composed of calcium, phosphorus, silicon and other elements. Similar to other silicate or phosphate systems, bioactive glass is an important representative of inorganic bioactive biological materials used in bone tissue engineering scaffolds. Its ion-soluble products (Si, Ca, P) can stimulate gene expression in osteoblasts [16, 17]. In addition, it has been reported that hollow mesoporous bioactive glass microspheres can be used as drug sustained-release carriers. Ding et al. improved the drug loading capacity of bone-implantable delivery systems by introducing hollow structure mesoporous bioactive glass spheres (HMBGs) through a sol–gel process. For in vitro drug release, HMBGs could sustain the storage and release of vancomycin hydrochloride (VAN) via a diffusion-controlled mechanism. Moreover, HMBGs incorporated with VAN provided a biomimetic microenvironment favored by cell adhesion and proliferation [18]. However, the composite scaffolds of polymer materials and inorganic nanoparticles without porogen are usually non-porous. The results of our research confirmed that the PLGA/BGP composite presented a loose and porous structure, while the PLGA/BG scaffold was solid with little porosity (Fig. S1(B)). The bioactive glass particles used in present research were prepared by the spray-drying method. As shown in Fig. 2, the BGPs were spherical. The inside of the spheroid was hollow. The shell of the spheroid was quite thin. The spheroids with this structure had a large specific surface area for drug loading. This was also proven by a protein adsorption and release assay. Besides, it is reported that, contrast with irregular bioactive glass, bioactive glass microspheres (BGM) in micrometer scale enhanced amorphous structure, optimized silicon release profiles and hydroxyapatite-forming bioactivity as well as improved biological properties, possessed the potential applications in drug delivery systems and scaffolds for tissue regeneration [19]. Based on the above reasons, spherical micron-scale BGP was chosen for this study.

Compared with chemosynthetic drugs, effective natural pharmaceutical ingredients with clear structures not only have good therapeutic effects but also have the advantages of few side effects and low drug resistance [20]. Therefore, the clinical applications of natural pharmaceutical ingredients are attracting increasing attention. Nef is a dibenzyl isoquinoline alkaloid extracted from *lotus* seed. It has been proven to have anti-experimental arrhythmia, antihypertensive, anti-myocardial ischemia, anti-platelet aggregation and anti-hydroxyl free radical effects. In addition, it also has anticancer potential. The effect of Nef on osteogenesis and osteoclasts was only recently reported. Chen et al. reported the inhibition of Nef on RANKL-induced osteoclast formation and promotion of it on the differentiation and bone mineralization activity of MC3T3-E1 cells [15]. These findings suggest that Nef is a potential signaling factor in bone tissue engineering. In the present study, Nef was chosen as the bioactive drug loaded in the composite. To determine the applied dosage of Nef, the proliferation and alkaline phosphatase (ALP) activity of MC3T3-E1 cells planted on composite membranes containing different Nef contents were detected. The results indicated that Nef promoted osteogenic differentiation and inhibited the proliferation of MC3T3-E1 cells in a content- and time-dependent manner. The composites containing less Nef had less effect on cell proliferation and osteogenic differentiation. With the increase in drug content, the toxicity of the composite to cells gradually strengthened. On the other hand, the increase in drug content promoted the osteogenic differentiation effect of the composite. However, when the drug content exceeded 25 μM, the promoting effect of the material on osteogenesis began to weaken. This result is consistent with Chen's report. At either the lowest concentration that inhibited proliferation or the concentration that promoted ALP activity the most, Chen's results were all lower than those in the present report. This is mainly attributed to the drug sustained release system established in this study. More drugs are loaded inside the complex and released slowly. It reduced the real-time drug concentration in the cell medium. However, this is beneficial for prolonging the biological activity of the material. The Nef can act stably on the defect area for a long time and avoid the side effects caused by repeated use.

As a component of bone tissue, in contrast with the osteogenesis of osteoblasts, osteoclasts play an important role in bone resorption. Osteoclasts can dissolve obsolete bone tissue and increase calcium concentrations in blood, further provide calcium and phosphorus for bone remodeling. However, excessive bone resorption induced by hyperactive osteoclasts can delay bone regeneration [21]. High expression of TRAP and CatK are the major markers of active osteoclasts. During bone resorption process, after attach to the surface of bone, osteoclasts secrete acids and proteases to create a lot of gaps between the bone matrix and themselves. TRAP is secreted into these gaps through exocytosis from the wavy margin of osteoclasts. Then The solid calcium phosphate minerals in the bone matrix are degrade by TRAP, together with other enzymes [22]. CatK is a cysteine protease with the highest expression and the strongest bone lytic activity in osteoclasts, and is a key enzyme in the process of bone resorption. Although it is reported that the CatK is needed in remodeling of bone defeat [23, 24]. The inhibition of CatK on bone marrow stem and progenitor cells differentiation in *vitro* should be remarked [25]. The bone regeneration greatly benefits from genetic deletion and chemical inhibitors of CatK, which abrogate bone resorption and stimulated periosteal bone remodeling [26]. Nef has recently been found to inhibit osteoclast differentiation and function [15]. The results observed in this study is consistent with the report. The composites with Nef not only inhibited the differentiation of RAW264.7 cells to osteoclast, but also downregulated TRAP activity. In addition, the expression and secretion of CatK was also downregulated. These results suggested that the composites with Nef obviously decelerated the bone resorption of osteoclasts. It is beneficial to the rapid bone healing of bone defects.

In addition to inhibiting osteoclasts, the composite also promoted osteogenesis. Col1 is a major component of the bone extracellular matrix [9]. In the early stages of bone repair, osteoblasts colonize the defect and begin to secrete extracellular matrix, including Col1, building a skeleton for osteoblast growth and mineralization. A large amount of early Col1 deposition will provide sufficient cell colonization and mineralization sites for bone repair. This process is the basis of bone repair and more important for bone regeneration than that in late stage, and is critical to evaluate the bone repair ability of materials. So that we test the expression of Col1 on day 3 and day 7. According to the present research, the composite of PLGA/BGP/25N promoted the expression of Col1 over a period of time. RUNX2 is a remarkable osteogenic transcription factor which mainly expressed in the early stage of osteogenic differentiation and involved in osteoblastic differentiation and skeletal morphogenesis and is essential for the maturation of osteoblasts and both intramembranous and endochondral ossification. The expression of RUNX2 is an important marker of osteoprogenitor cell differentiation into osteoblasts [27]. In RunX2-mediated osteogenic differentiation, cells transmit signals to the nucleus to direct the expression of RUNX2, firstly. After that, cells undergo osteogenic differentiation under the intracellular action of RUNX2 and express other osteogenesis-related proteins (OPN, OCN, et.al). So that we tested the expression of RUNX2 on day 3 and day 7. As reported in this study, the expression of RUNX2 was upregulated by composites containing Nef in the early stage. This indicated that the composite containing Nef is beneficial to osteogenic differentiation. OPN is a glycosylated protein widely distributed in the extracellular matrix. It is an important bone matrix protein that is closely related to bone formation and development. OCN is a vitamin K-dependent calcium-binding protein. It is mainly synthesized by osteoblasts, odontoblasts, and some by proliferative chondrocytes and plays an important role in regulating bone calcium metabolism. In bone repair, the OCN is mainly associated with bone mineralization, which occurs after the deposition of extracellular matrix. This process usually takes place in late and very late stages of cell culture. So that we test the expression of OCN on day 7 and day 14 [28]. The results showed that Nef-containing composites upregulated the expression of OPN and OCN in cells at the mid and late stages, revealing the potential for promoting bone repair.

According to the results, the effect of Nef-containing composites on osteoclast inhibition and osteogenesis promotion was confirmed. However, the mechanism still needs to be studied. PI3K/AKT/mTOR is a classical axis in osteogenic differentiation. Hui Liu et al. investigated the mechanism of morroniside in preventing bone loss, and found that morroniside could promote osteoblast differentiation by activating PI3K/AKT/mTOR pathway, thereby treating osteoporosis [29]. In addition, mTORC1, a typical mTOR, has emerged as a common effector mediating the bone anabolic effect of IGF-1. The IGF-1 stimulates osteoblastic differentiation of mesenchymal stem cell (MSCs) by activating mTOR [30]. It has been reported that Nef can activate the IGF-1R/PI3K/AKT/mTOR pathway in H9C2 cardiomyocytes [14]. The present study confirmed for the first time that the same effect was also observed in osteoblast MC3T3-E1 cells. This pathway is closely related to IGF-1-mediated osteogenic differentiation.

It has been reported that Nef can inhibit osteoclast differentiation by inhibiting the NF-κB pathway at the early stage of cell culture (1–3 days) but not at the intermediate stage (3–5 days) or late stage (5–7 days) [15]. In this study, osteoclasts cultured for 7 days were collected, and related proteins were detected. NFATC1, a transcription factor related to osteoclast differentiation, is expressed in the early or middle stages of osteoclast differentiation in vitro. However, high expression level of NFATC1 in late stage still enhance osteoclastogenesis [31]. Day 7 is still an early stage in the whole process of bone repair. Excessive bone resorption should be taken seriously. The results showed that the NFATC1 level was up-regulated in the PLGA/BGP group, and the sustained release of Nef could further inhibit the expression of NFATC1 by inhibiting the NF-κB pathway of osteoclasts at 7 days, thus inhibiting osteoclast differentiation at the late stage in *vitro.* The effect duration of Nef has been extended. This will inhibit the excessive bone resorption in the early stage of bone repair. According to the previous results, BGP degraded either in Si-rich remnants or in CaP-shells. Osteoclast-like cells developed exclusively on resorbable substrates. High calcium and phosphorus environment can activate osteoclast differentiation [32]. However, control-released Nef from the composite can effectively inhibit osteoclasts by inhibiting the NF-κB pathway in the long term. Meanwhile, bioactive glass can stimulate the secretion of IGF-1 by osteoblasts, while Nef can effectively activate the IGF-1R/PI3K/AKT/mTOR pathway to enhance IGF-1-mediated osteogenic differentiation.

According to the in *vitro* experiments, the bone regeneration potential of the material was demonstrated. To further verify the effect of the material on bone regeneration in *vivo*, a rat skull defect model was used in this study. The experimental results showed that compared with the other three groups, the PLGA/BGP/Nef group could more effectively promote the formation and maturation of new bone in the defect site and form a complete bony connection to cover the defect site. Different from some other reports, the PLGA/BGP group did not have a significant advantage in bone-promoting repair compared with the PLGA group. This is perhaps because bioglass particles were mostly transformed in CaP-shells, then being resorbable. The occurrence and number of osteoclast-like cells was correlated to the resorbability of the underlying substrate. High calcium and phosphorus environment activate osteoclast differentiation [30]. These osteoclasts provide raw materials for bone repair by phagocytosis and decomposition of BGP and surrounding bone tissue but also delay the initial bone repair. However, collagen fibers surrounding the material in PLGA/BGP group were significantly denser than those in the PLGA group. And the mature bone in PLGA/BGP group was also more than that in PLGA group. Over time, these collagen fibers are likely to serve as anchors for new bone mineralization. At that time, the bone repair advantages of bioactive glass will be realized.

## Conclusions

In this study, a PLGA/BGP/Nef composite porous material with a special radial pore structure was prepared by the phase exchange method for the first time, in which Nef was controllably released. The composite was demonstrated to effectively inhibit osteoclasts and promote osteogenesis. The underlying mechanisms were clarified. The sustained release of Nef in the PLGA/BGP/Nef composite could downregulate the expression of NFATC1 by inhibiting the NF-κB pathway to restrain osteoclasts and effectively activate the IGF-1R/PI3K/AKT/mTOR pathway to enhance IGF-1-mediated osteogenic differentiation. The results of animal experiments show that the material can effectively promote bone injury repair and has the potential for clinical application.

## Supplementary Information

**Additional file 1.**

## Data Availability

Please contact author for data requests.
