# Supplementary material for: PLGA/BGP/Nef porous composite restrains osteoclasts by inhibiting the NF-κB pathway, enhances IGF-1-mediated osteogenic differentiation and promotes bone regeneration
Source: J Biol Eng. 2023 Jul 17;17:45. doi: 10.1186/s13036-023-00354-8 (PMC10353098; doi:10.1186/s13036-023-00354-8)
Supplement: Supplementary file 1 — Additional file 1. [file 13036_2023_354_MOESM1_ESM.docx]

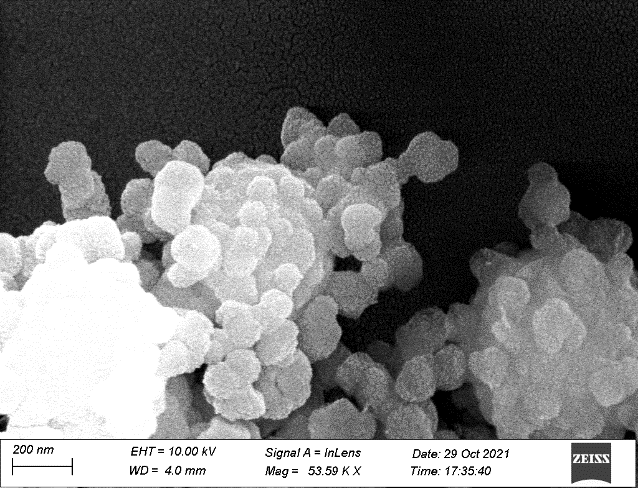

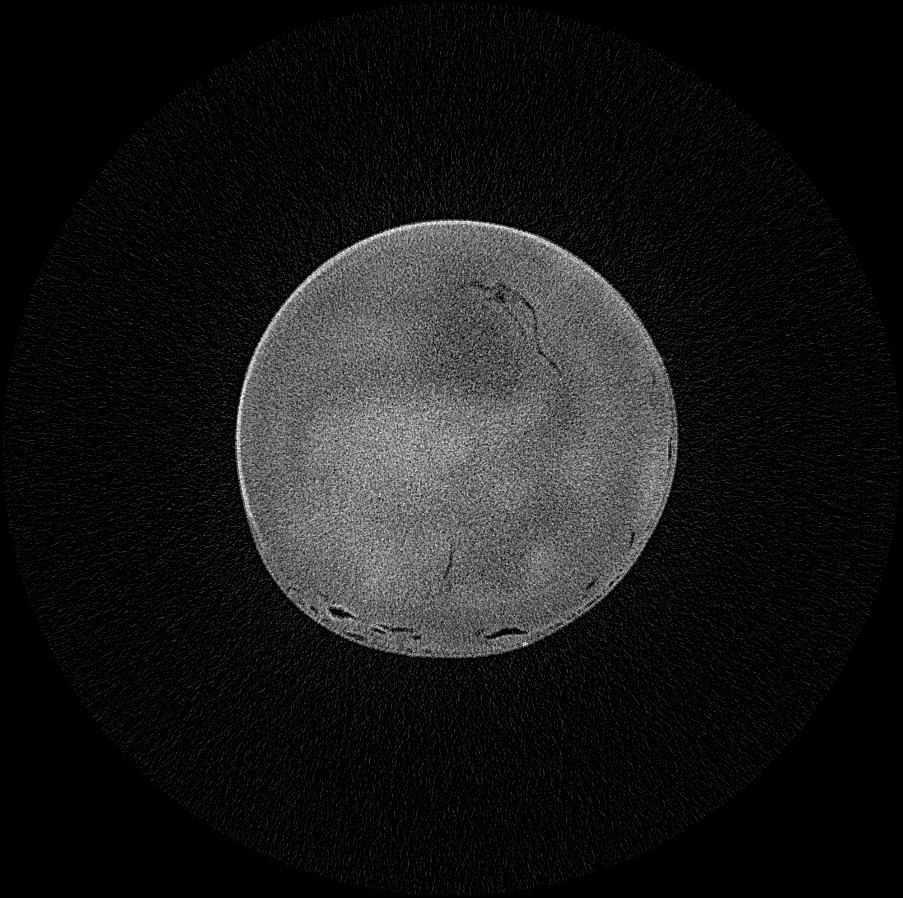


**Fig.S1** (A)SEM photo of bioactive glass particles (BG) prepared by sol-gel method and grinding. Bar=100 nm (B) The single layer scan of PLGA/BG composite based on micro-CT results. Bar=1 mm.

**Fig.S2** The FT-IR spectra of the composites between 3240 nm and 3300 nm.


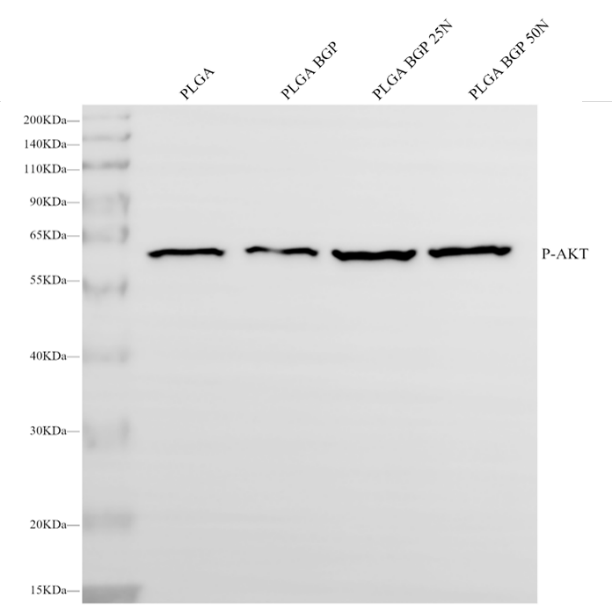

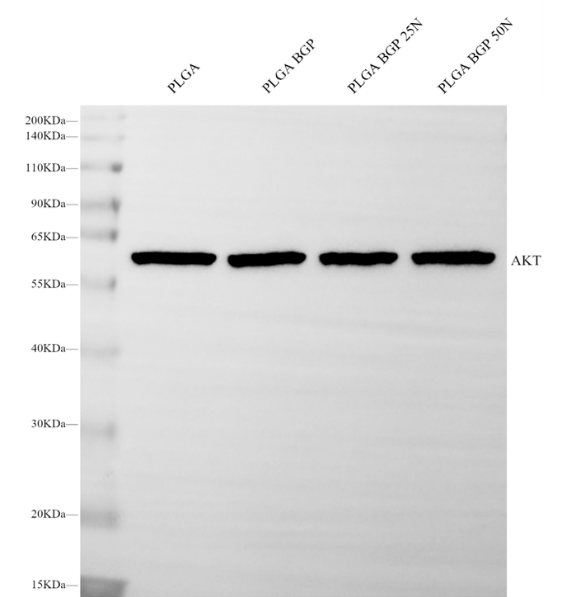

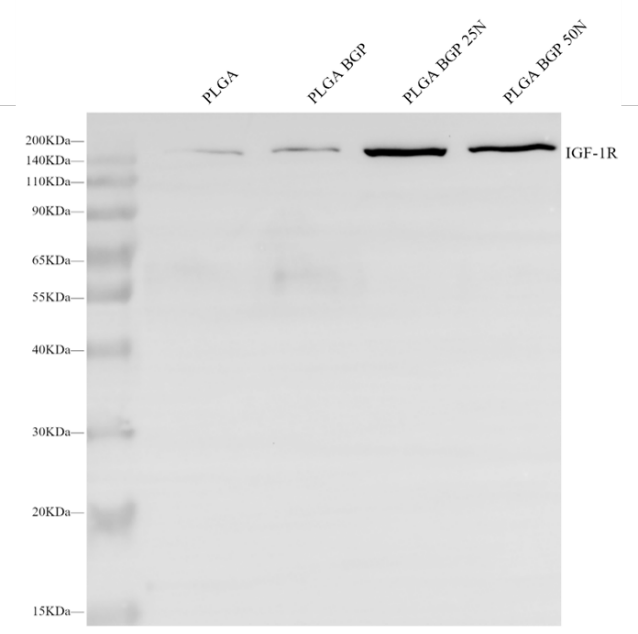

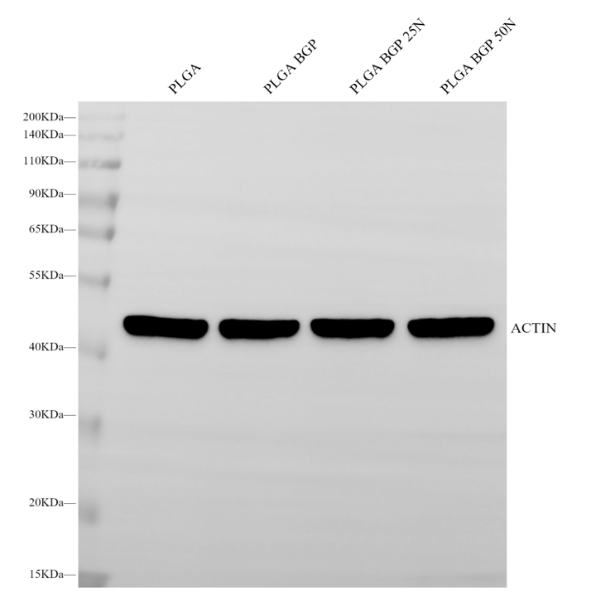

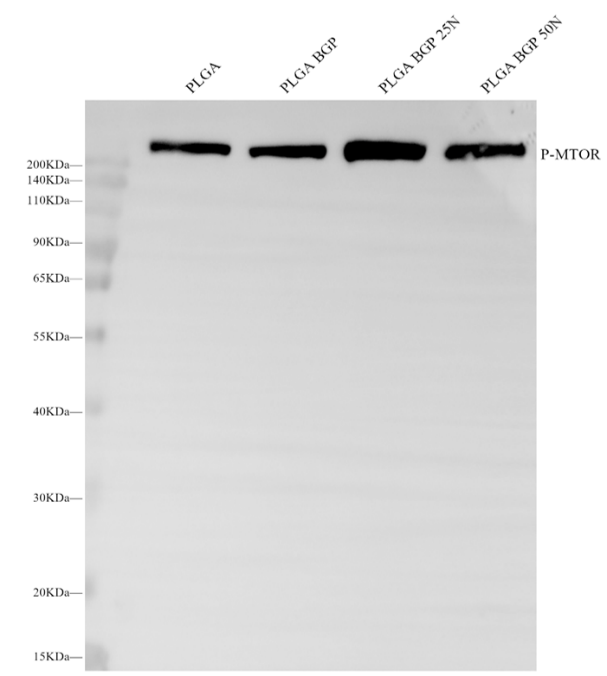

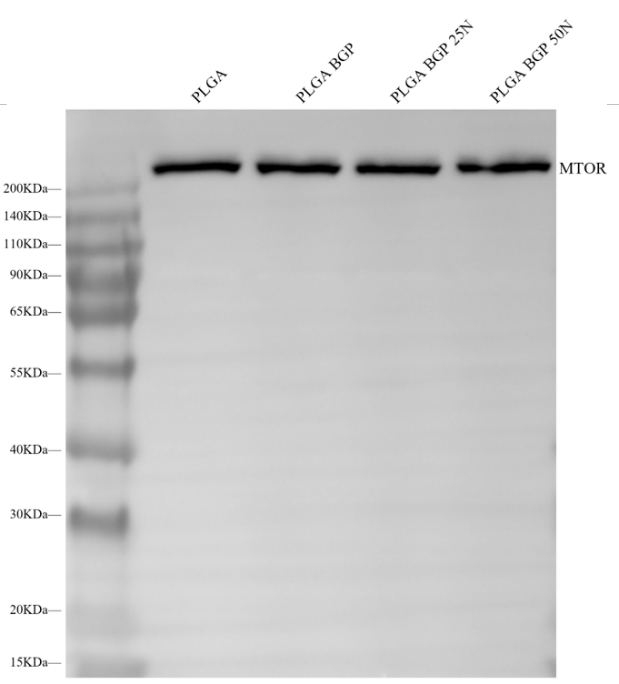

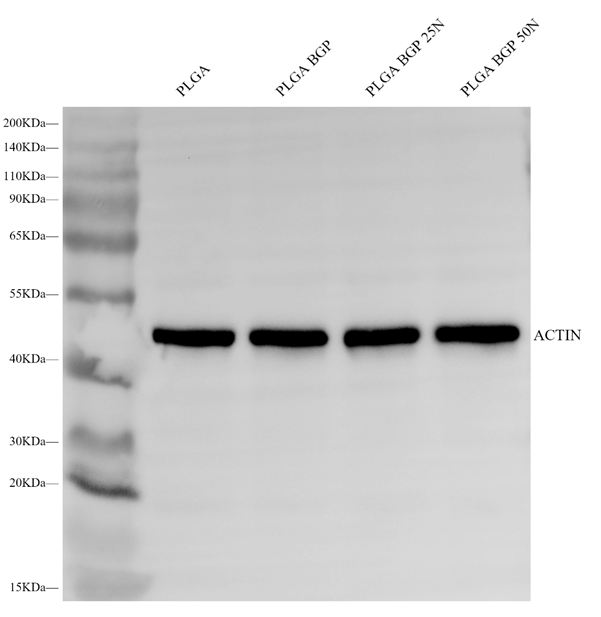

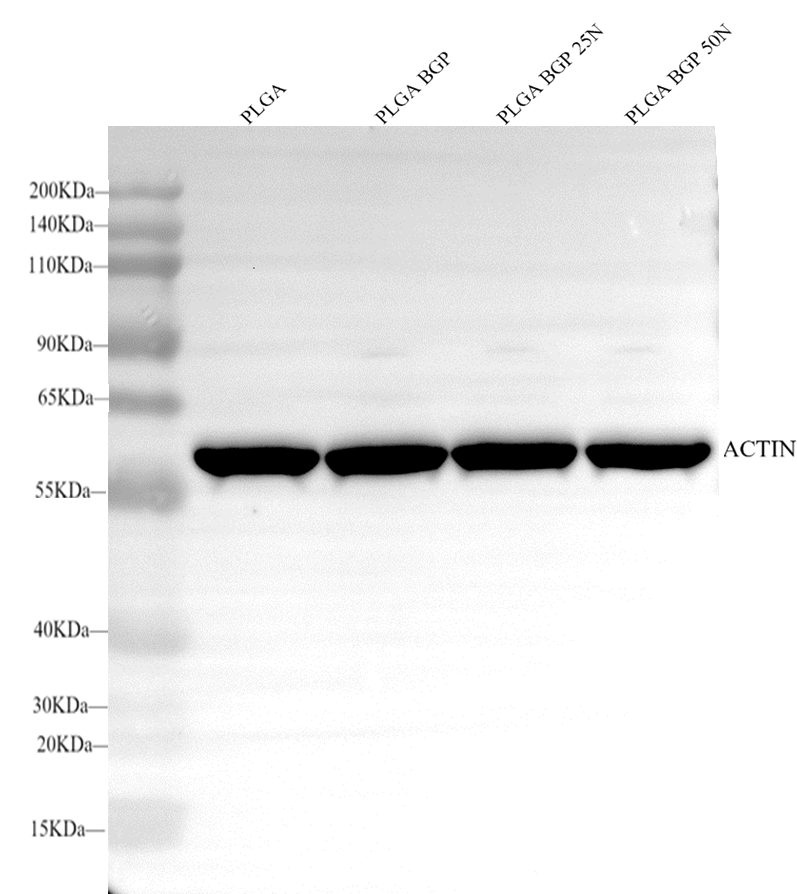

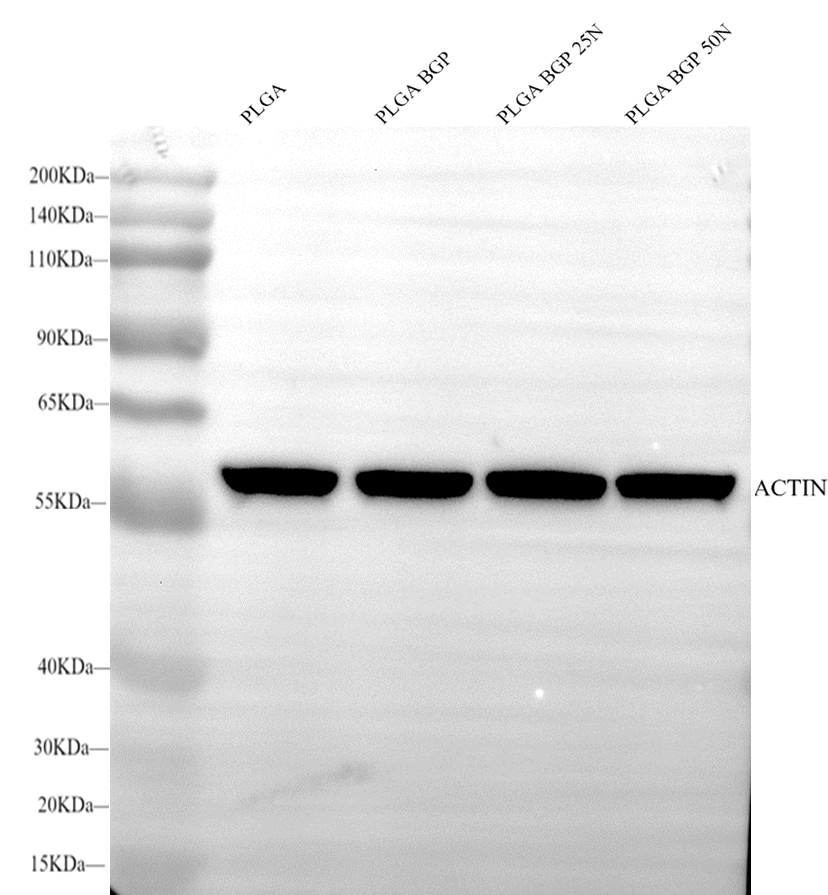

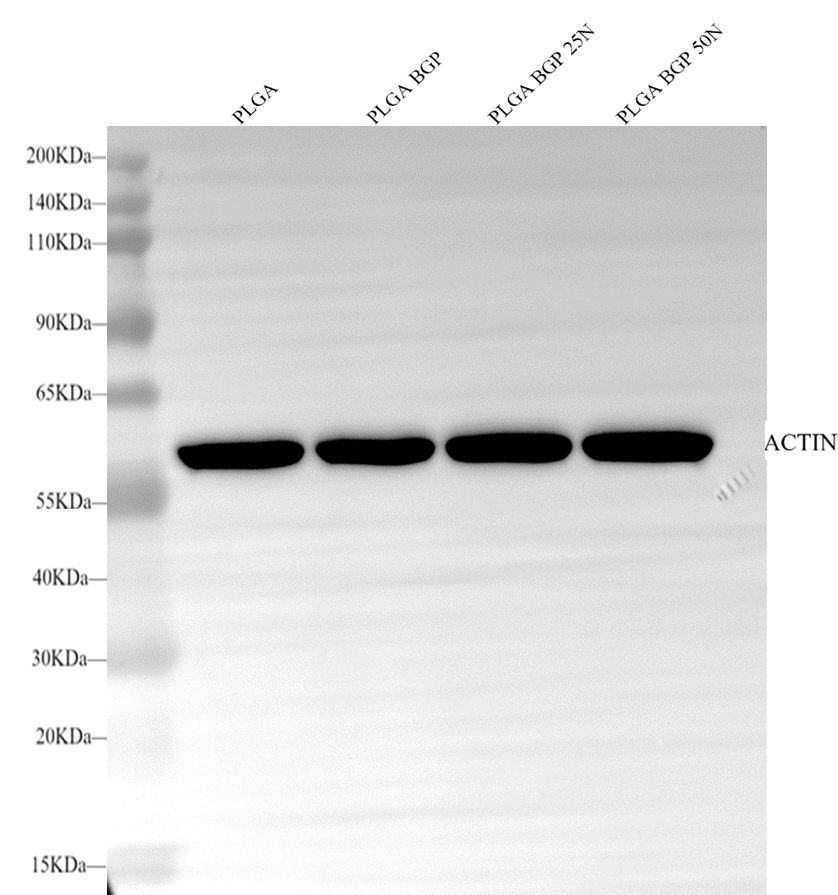


**Fig.S3** full membrane images of all westernblot in Fig. 7(A).


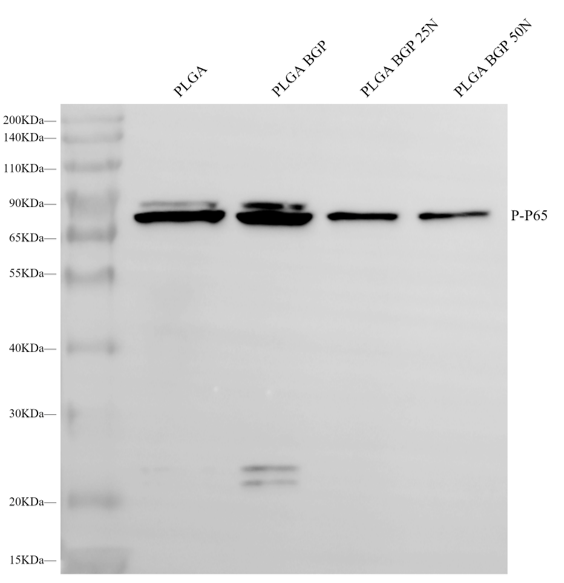

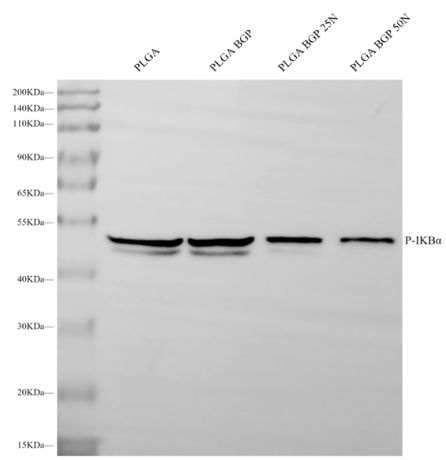

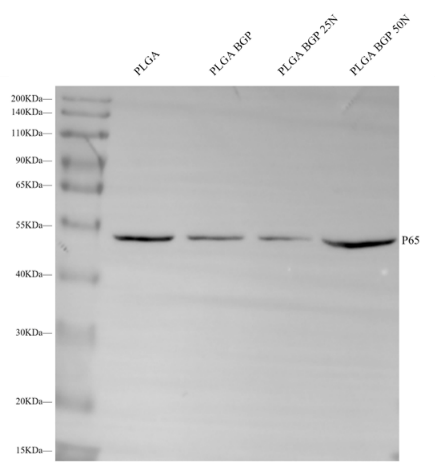

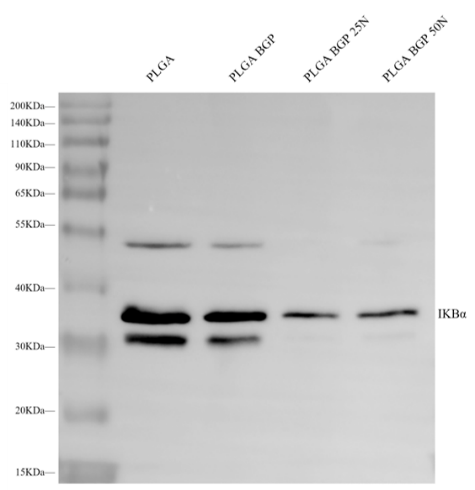

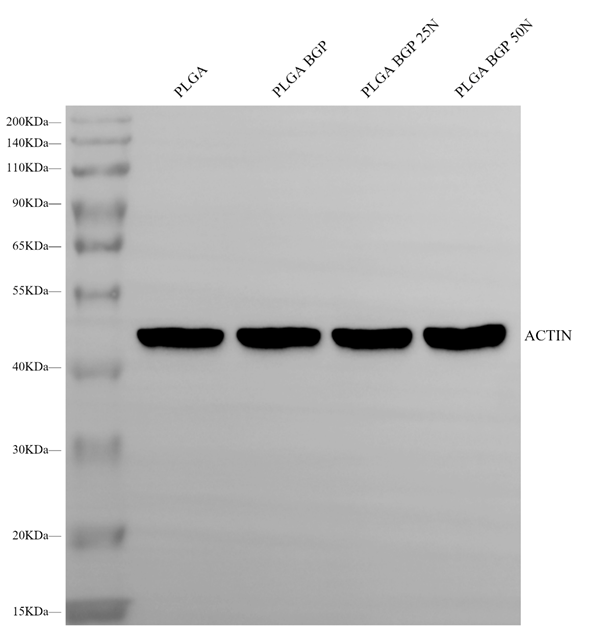

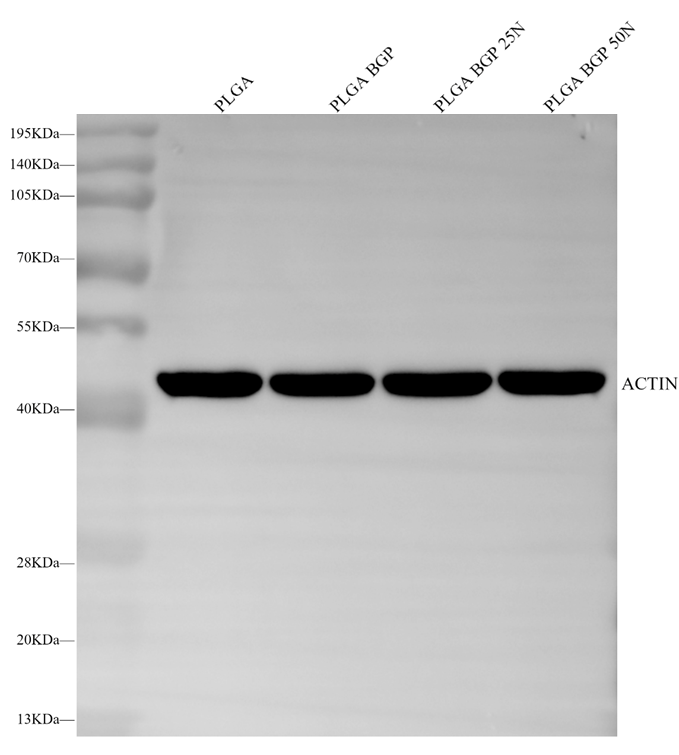

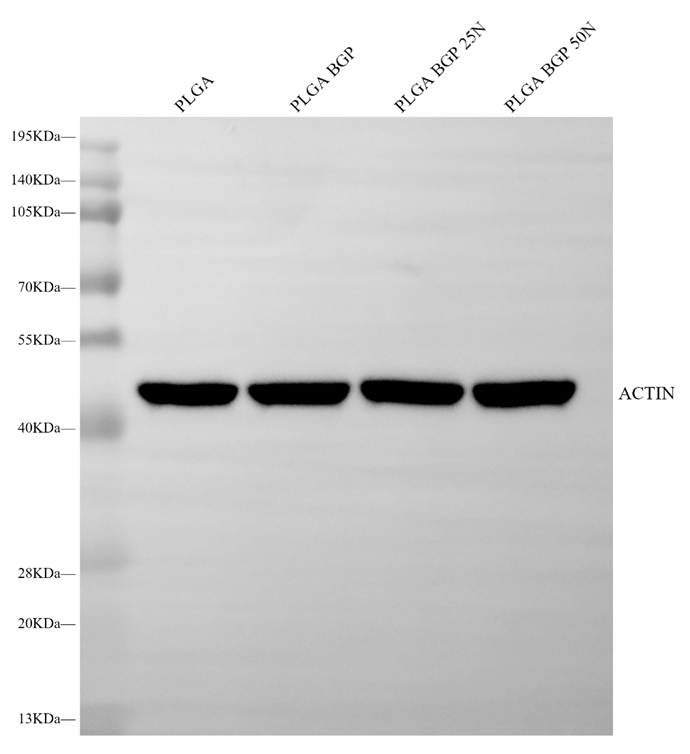

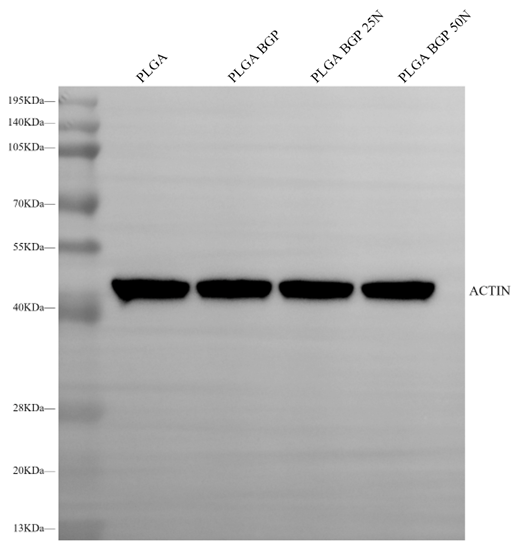


**Fig.S4** full membrane images of all westernblot in Fig. 9(A).


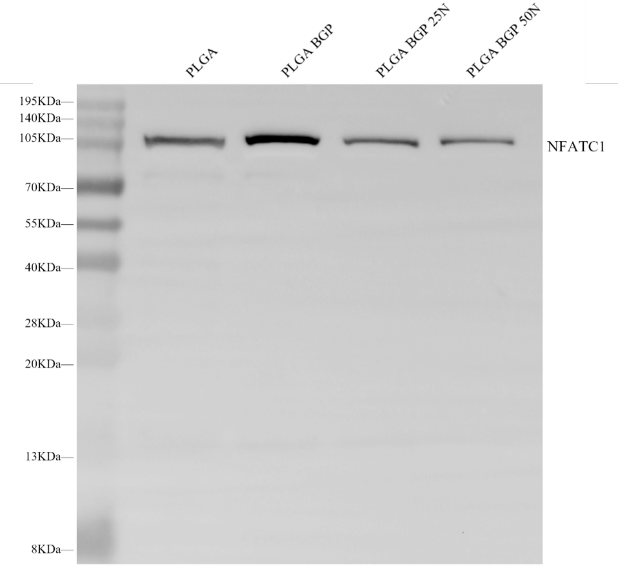

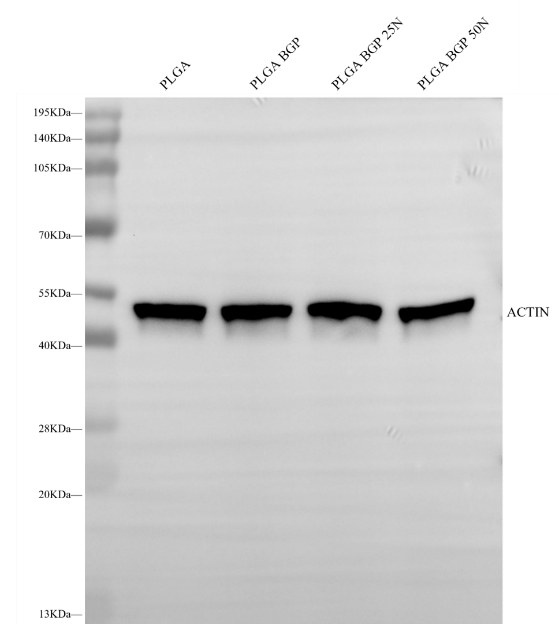


**F****ig.S5** full membrane images of all westernblot in Fig. 9(C).

**Fig. S6** The standard curve of ELISA kit.


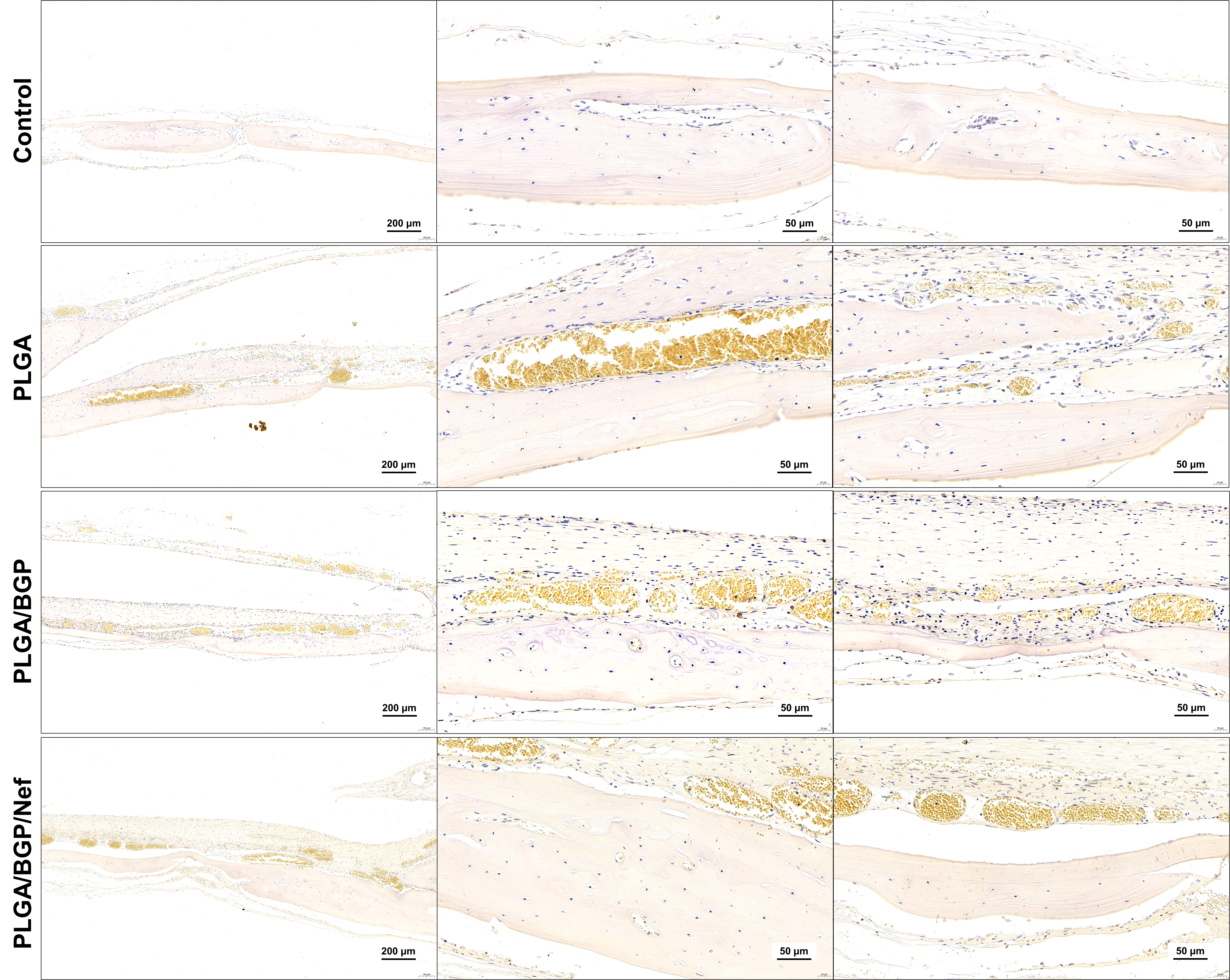


**Fig.S7** The TRAP staining results in rat calvarial bone defects.

| **Name** | **Brand** | **Product code** | **Host species** | **Dilution ratio** |
| --- | --- | --- | --- | --- |
| ACTIN | Servicebio | GB12001 | Mouse | 1: 2000 |
| IGF-1R | BIOSS | BS-4985R | Rabbit | 1: 1000 |
| AKT | Servicebio | GB111114 | Rabbit | 1: 1000 |
| p-AKT | Affinity | AF0908 | Rabbit | 1: 1000 |
| mTOR | Servicebio | GB111840 | Rabbit | 1: 3000 |
| p-mTOR | Servicebio | GB111839 | Rabbit | 1: 1000 |
| P65 | Servicebio | GB12142 | Mouse | 1: 1000 |
| p-P65 | Servicebio | GB113882 | Rabbit | 1: 1000 |
| IKBα | Servicebio | GB111509 | Rabbit | 1: 1000 |
| p-IKBα | AFFINITY | AF2002 | Rabbit | 1: 1000 |
| NFATC1 | abcam | ab2796 | Mouse | 1: 2000 |

**Table S1** Information of westernblot primary antibodies
